# HLA-C downregulation by HIV-1 adapts to host HLA genotype

**DOI:** 10.1371/journal.ppat.1007257

**Published:** 2018-09-04

**Authors:** Nathaniel D. Bachtel, Gisele Umviligihozo, Suzanne Pickering, Talia M. Mota, Hua Liang, Gregory Q. Del Prete, Pramita Chatterjee, Guinevere Q. Lee, Rasmi Thomas, Mark A. Brockman, Stuart Neil, Mary Carrington, Bosco Bwana, David R. Bangsberg, Jeffrey N. Martin, Esper G. Kallas, Camila S. Donini, Natalia B. Cerqueira, Una T. O’Doherty, Beatrice H. Hahn, R. Brad Jones, Zabrina L. Brumme, Douglas F. Nixon, Richard Apps

**Affiliations:** 1 Department of Microbiology, Immunology & Tropical Medicine, The George Washington University, Washington DC, United States of America; 2 Faculty of Health Sciences, Simon Fraser University, Burnaby, Canada; 3 Department of Infectious Disease, King’s College London School of Medicine, Guy’s Hospital, London, United Kingdom; 4 Department of Statistics and Biostatistics, George Washington University, Washington DC, United States of America; 5 AIDS and Cancer Virus Program, Frederick National Laboratory for Cancer Research sponsored by the National Cancer Institute, Frederick, Maryland, United States of America; 6 Cancer and Inflammation Program, HLA Immunogenetics Section, Basic Science Program, Frederick National Laboratory for Cancer Research sponsored by the National Cancer Institute, Frederick, Maryland, United States of America; 7 Ragon Institute of Massachusetts General Hospital, Massachusetts Institute of Technology and Harvard University, Cambridge, Massachusetts, United States of America; 8 US Military HIV Research Program, Walter Reed Army Institute of Research, Silver Spring, Maryland, United States of America; 9 Henry M. Jackson Foundation, Bethesda, Maryland, United States of America; 10 British Columbia Centre for Excellence in HIV/AIDS, Vancouver, Canada; 11 Mbarara University of Science and Technology, Mbarara, Uganda; 12 Oregon Health & Science University, Portland State University School of Public Health, Portland, Oregon, United States of America; 13 Department of Epidemiology and Biostatistics, University of California, San Francisco, San Francisco, California, United States of America; 14 University of Sao Paulo, Sao Paulo, Brazil; 15 Departments of Medicine and Microbiology, University of Pennsylvania, Philadelphia, Pennsylvania, United States of America; University of Wisconsin, UNITED STATES

## Abstract

HIV-1 can downregulate HLA-C on infected cells, using the viral protein Vpu, and the magnitude of this downregulation varies widely between primary HIV-1 variants. The selection pressures that result in viral downregulation of HLA-C in some individuals, but preservation of surface HLA-C in others are not clear. To better understand viral immune evasion targeting HLA-C, we have characterized HLA-C downregulation by a range of primary HIV-1 viruses. 128 replication competent viral isolates from 19 individuals with effective anti-retroviral therapy, show that a substantial minority of individuals harbor latent reservoir virus which strongly downregulates HLA-C. Untreated infections display no change in HLA-C downregulation during the first 6 months of infection, but variation between viral quasispecies can be detected in chronic infection. Vpu molecules cloned from plasma of 195 treatment naïve individuals in chronic infection demonstrate that downregulation of HLA-C adapts to host HLA genotype. HLA-C alleles differ in the pressure they exert for downregulation, and individuals with higher levels of HLA-C expression favor greater viral downregulation of HLA-C. Studies of primary and mutant molecules identify 5 residues in the transmembrane region of Vpu, and 4 residues in the transmembrane domain of HLA-C, which determine interactions between Vpu and HLA. The observed adaptation of Vpu-mediated downregulation to host genotype indicates that HLA-C alleles differ in likelihood of mediating a CTL response that is subverted by viral downregulation, and that preservation of HLA-C expression is favored in the absence of these responses. Finding that latent reservoir viruses can downregulate HLA-C could have implications for HIV-1 cure therapy approaches in some individuals.

## Author summary

HLA-C is a member of the major histocompatibility complex class-I (MHC-I) family of molecules which are integral to many responses of innate and adaptive immunity. HIV-1 can downregulate the expression level of HLA-C on infected cells, using the viral protein Vpu, but the magnitude of HLA-C downregulation varies widely between primary HIV viruses. This provides an opportunity to identify opposing pressures on HLA-C expression in infected human individuals. We find that viral downregulation of HLA-C associates with both host and virus genotype, defining allelic differences in HLA-C and Vpu that will help to identify the specific immune responses which result in viral downregulation or preservation of HLA-C. These responses could represent candidates for immune therapy, given their demonstrated effects in vivo. We also find that HIV-1 viruses from the latent reservoir of some individuals can downregulate HLA-C, indicating that in certain individuals HIV-1 cure will require the removal of virus that is able to downregulate HLA-C. The unique role of HLA-C, exerting selection pressures that result in variable modulation of its expression, may make it possible for immunotherapies to achieve enhanced efficacy if both of the opposing pressures on HLA-C expression could be established in the same individual.

## Introduction

Human Leukocyte Antigen class-I (HLA-I) molecules present peptides from intracellular proteins at the cell surface. Classical HLA-I molecules are highly polymorphic and expressed from three loci, HLA-A,—B and—C, which differ in some respects. Polymorphism is most pronounced for HLA-B, and HLA-A/B are expressed at around 10-fold higher levels than HLA-C at the cell surface [1,2]. In the event of HIV-1 infection, HLA-A/B/C proteins can all present viral peptides which are recognized by CD8+ T cells, and result in the triggering of effector responses including target cell killing. Several lines of evidence provide unambiguous support for a critical role of CD8+ T cell responses in partial control of HIV-1 infection: the emergence of cytotoxic T lymphocytes (CTL) coincides with reduction in viral load after acute infection [3–5], HLA-I alleles associate with outcomes of HIV-1 infection or viral sequence adaptation [6–9], and CTL can eliminate infected cells in vitro [10]. Many pathogens evade CTLs by disrupting HLA expression, but this can incur recognition by innate immune cells. Natural Killer (NK) cells are regulated by inhibitory receptors for self HLA-I molecules, such as inhibitory killer immunoglobulin-like receptors (KIR). Cells with decreased HLA expression fail to ligate these inhibitory receptors, resulting in NK activation and cytotoxicity [11]. Associations between KIR alleles and viral load provide evidence that NK cells can influence the outcome of HIV-1 infection in vivo, and NK cells can be observed to respond to HIV-1 infected cells in vitro [12–14].

Faced with these challenges, HIV-1 demonstrates sophisticated manipulation of HLA-I molecules, in which both the locus specificity and magnitude of downregulation appears to be important. It has long been known that HLA-A/B, but not HLA-C molecules, are downregulated by the Nef protein of HIV-1 [15–17]. Downregulation of HLA-A/B is well-established to subvert CTL mediated host immunity in vitro and in vivo [18,19]. The clinical importance of Nef is confirmed by rare forms of HIV-1 that lack part of Nef, which do not cause AIDS when infecting humans [20,21]. Although downregulated by around 5-fold on infected primary cells, HLA-A/B are not removed entirely from the surface of infected cells [2]. This may maintain some inhibition of NK cells, although other receptor-ligand interactions during infection can also clearly influence NK cell responses [22–25].

We recently found that HLA-C can be downregulated by the Vpu protein of HIV-1, and this observation has already been replicated by 3 independent groups [26–29]. Vpu is necessary and sufficient for HLA-C downregulation, but Vpu does not affect HLA-A/B. Downregulation of HLA-C by Vpu occurs in primary cells infected with primary HIV-1 clones. Transmitted/founder viruses, viruses from chronic infection, and representatives of all subtypes of HIV-1 are capable of downregulating HLA-C [26]. This supports a biologically relevant role for downregulation of HLA-C by HIV-1, and the finding is consistent with evidence accumulated over the past decade for a role of HLA-C expression level in HIV-1 disease. Early genome-wide association studies identified a strong effect of a variant marking HLA-C in the outcome of HIV-1 infection [8,9]. Unlike HLA-A/B for which individual alleles associate with HIV-1 viral load, associations at the HLA-C locus implicated large groups of alleles. It was found that host HLA-C alleles vary in expression level under normal conditions, and that HLA-C expression level as a continuous variable correlated inversely with HIV-1 viral load [30,31]. Higher HLA-C expression may be disadvantageous for the virus due to more potent CTL responses, as both HIV-specific CTL and viral escape mutation associate more strongly with HLA-C alleles that are expressed at higher levels [31,32].

A key difference between HLA-C downregulation by Vpu and the modulation of HLA-A/B by Nef, is that the former varies much more frequently between HIV-1 viruses. Indeed, downregulation of HLA-C by HIV-1 had escaped detection for so long because the widely-studied lab-adapted strain NL4-3 does not downregulate HLA-C. In the initial report of HLA-C downregulation by Vpu, 15 infectious molecular clones showed a continuous distribution in ability to downregulate HLA-C, in contrast to almost identical levels of HLA-A downregulation [26]. A comprehensive screen of 360 primary Nef variants found some variability in downregulation of HLA-A, but that the magnitude of this function was on average well-preserved [33]. This suggests that a similar level of HLA downregulation provides optimal evasion of HLA-A/B mediated immunity across individuals. Responses that target HLA-C differ much more between individuals in the selection pressure they exert on HLA expression level. Targeting of HLA-A/B and HLA-C by separate viral proteins, and their contrasting patterns of downregulation between individuals, emphasizes differences in the biological role of these HLA molecules in HIV-1 infection.

The specific selection pressures which favor viral downregulation of HLA-C in some individuals, but HLA-C preservation in others, are not known. In contrast to HLA-A/B, the physiological significance of HLA-C restricted CTL is less clear. HLA-C restricted CTL responses can be detected, and viral escape mutations associating with HLA-C alleles are observed [34–38]. However, these mutations are not necessarily deleterious for the virus, and unlike for HLA-A/B individual HLA-C alleles do not strongly associate with HIV-1 viral load [7,39]. Viral downregulation of HLA-C is also more likely than downregulation of HLA-A/B, to increase infected cell susceptibility to NK cell cytotoxicity. This is because inhibitory KIR2DL receptors which recognize self HLA-C alleles are present in all individuals, unlike KIR3DL which bind only a subset of HLA-A/B allotypes that are absent in many individuals [40]. In vitro experiments have provided evidence consistent with HLA-C downregulation inhibiting CTL but activating NK cells. An HLA-C*03 restricted CTL clone, which recognizes an Env epitope, showed decreased inhibition of an NL4-3 virus mutated so that its Vpu was able to downregulate HLA-C [26]. KIR2DL2+ NK cells showed increased inhibition of the virus JR-CSF compared to its mutant lacking Vpu [27]. In both of these in vitro approaches, the small effect and analysis of single epitopes or effectors limits generalization to a physiological role of HLA-C downregulation in CTL or NK function. The variation in HLA-C downregulation between primary HIV-1 viruses provides an opportunity to identify the selection pressures acting on HLA-C expression in human individuals.

To identify what leads to differential HLA-C downregulation by virus from different infections, it is necessary to expand the number and type of primary viruses for which this phenotype has been studied. To date 15 infectious molecular clones and viruses from six transmission pairs, have been measured for ability to downregulate HLA-C [26,27,29]. This study characterizes HLA-C downregulation by 128 viruses from the latent reservoir of 19 anti-retroviral therapy (ART)-suppressed individuals, 41 samples from 9 individuals with untreated infection, and cloned Vpu molecules from a further 195 chronically infected individuals spanning four viral subtypes. Adaptation of HLA-C downregulation to host genotypic variants is identified, that indicates selection pressures involved in the viral manipulation of HLA-C.

## Results

### HIV-1 reservoir viruses can downregulate HLA-C

**Fig 1:**
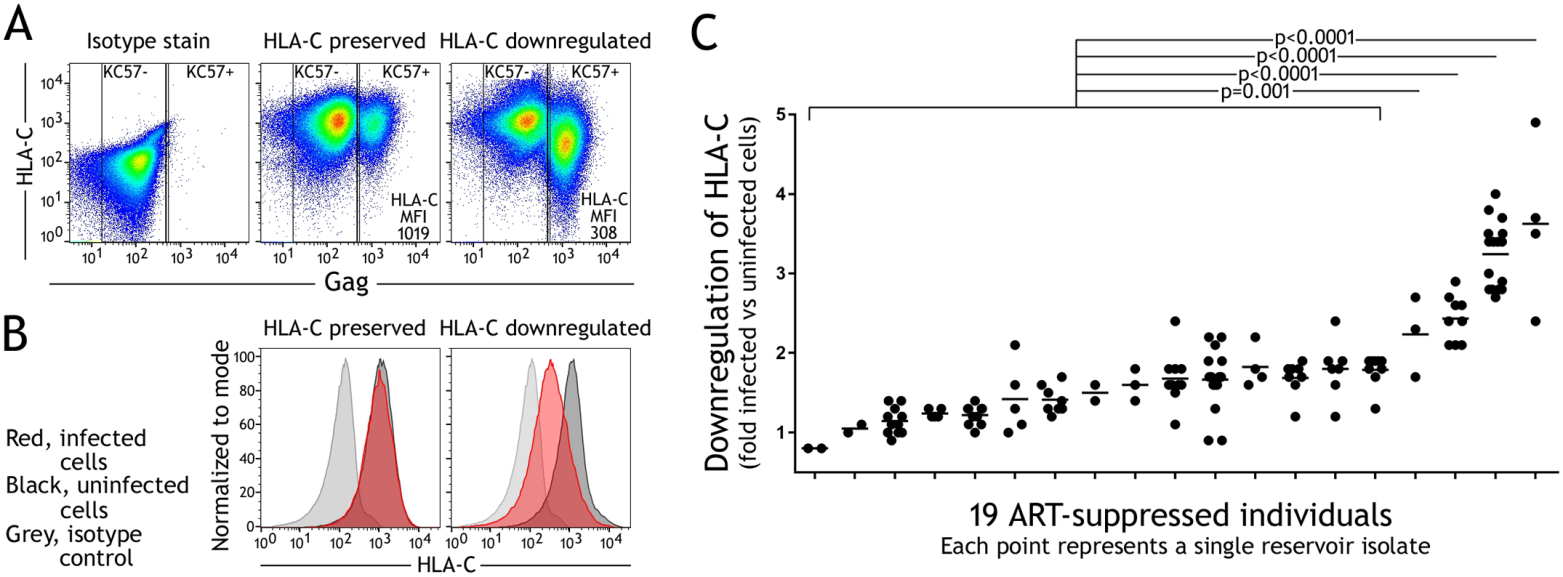
Inducible infectious HIV-1 reservoir viruses can downregulate HLA-C. (A) Viral isolates were generated from ART-suppressed individuals and infected co-cultured Molt-4 cells, where HLA-C downregulation was determined by comparing HLA-C staining between uninfected and infected cells, discriminated by intracellular staining for Gag. (B) Representative staining of Molt-4 cells in cultures infected with viral isolates that preserve or downregulate HLA-C. HLA-C staining is shown for infected (red) and uninfected cells (black) compared to an isotype control stain (grey). (C) HLA-C downregulation observed for 128 isolates from 19 individuals. Statistical analyses are unpaired t tests.

In virally suppressed individuals adhering to effective ART, a reservoir of infected cells results in viral rebound if ART is discontinued [41]. This viral reservoir is the subject of intense interest as a barrier to the cure of HIV-1 [42]. We have characterized the ability to downregulate HLA-C for 128 inducible infectious HIV-1 isolates, from peripheral blood of 19 ART-suppressed individuals. Cultures were generated for quantitative viral outgrowth assays (QVOA), with limiting dilutions of donor CD4+ T lymphocytes stimulated with PHA, and induced viral isolates infected co-cultured Molt-4 T cells [43]. In these conditions viral isolates represent clonal outgrowths with negligible mutation in the two weeks of in vitro culture [44]. HLA-C downregulation by each viral isolate was quantified by comparing flow cytometry staining of HLA-C, between infected and uninfected Molt-4 cells within the same culture well (Fig 1A). Isolates varied widely in their ability to downregulate HLA-C, ranging from no effect to robust downregulation (Fig 1B). For viruses showing the strongest downregulation of HLA-C, this was of a magnitude comparable to that observed for the downregulation of HLA-B when primary CD4+ T cells are infected with HIV-1 in vitro [2,45]. 19 individuals were studied and of these, the viral isolates of 15 individuals demonstrated no or low ability to downregulate HLA-C, whereas in 4 individuals we observed downregulation of HLA-C by multiple viral isolates (Fig 1C).

### HLA-C downregulation in untreated HIV-1 infection

**Fig 2:**
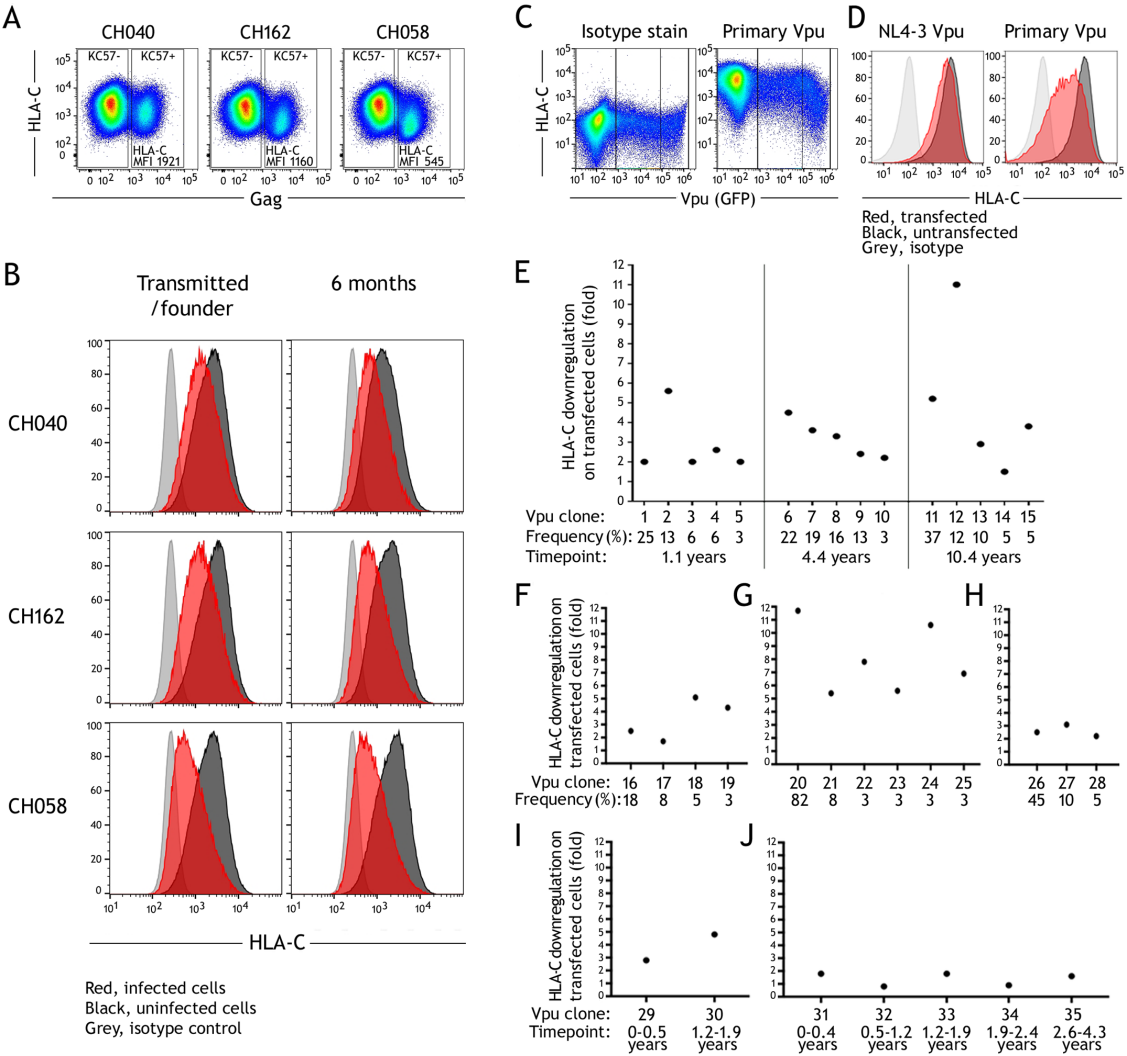
HLA-C downregulation in untreated HIV-1 infection. (A) Infectious molecular clones were assessed for ability to downregulate HLA-C for 3 individuals: CH040, 162, 058. Representative staining is shown for primary CD4+ cells infected in vitro, with HLA-C staining compared between infected and uninfected cells within a culture. (B) For the 3 individuals infectious molecular clones of both transmitted/founder viruses and those 6 months after infection were tested. HLA-C staining is shown for infected (red) and uninfected cells (black) compared to an isotype control stain (grey). (C) For ART-naïve individuals multiple cloned Vpu molecules were measured for ability to downregulate HLA-C by transfection of HeLa cells, with transfected cells in a culture identified by co-transfection of a GFP expression plasmid. (D) Staining for HLA-C is shown for Vpu clones with differential downregulation, showing transfected (red) and untransfected cells (black) compared to an isotype control (grey). (E) HLA-C downregulation is shown for 15 Vpu clones from one individual sampled at 3 timepoints over 10 years. (F-H) HLA-C downregulation observed for multiple clones from a single timepoint for 3 individuals. (I,J) HLA-C downregulation observed for dominant Vpu clones from longitudinal timepoints for 2 individuals.

Given the variation in HLA-C downregulation between primary viruses from different individuals and between multiple viral isolates from the reservoir of an individual (Fig 1C) [26], we investigated if HLA-C downregulation changed longitudinally during early untreated infection. Using previously described paired primary infectious molecular clones from individuals, we compared HLA-C downregulation between a transmitted/founder virus and a representative virus from the same individual 6 months after infection [46,47]. Primary CD4+ T cells were infected in vitro and HLA-C downregulation determined by flow cytometry, comparing HLA-C staining on infected versus uninfected CD4 cells within the same culture well (Fig 2A). For 3 individuals studied, there was no difference in the HLA-C downregulation by the transmitted compared to 6 month virus. This included viruses with HLA-C downregulation which was weak (CH040), moderate (CH162), and strong (CH058) (Fig 2B).

To investigate HLA-C downregulation later in infection, we studied cloned Vpu molecules generated from the plasma of 6 untreated individuals. Cloned Vpu genes were expressed by transfection in HeLa cells using a Rev-dependent vector, and HLA-C downregulation quantified by flow cytometry comparing staining of transfected versus untransfected cells within a culture well (Fig 2C and 2D). Vpu-mediated downregulation of HLA-C measured by transfection of HeLa cells, correlates robustly with HLA-C downregulation by primary HIV-1 viruses (S1 Fig). For one donor sampled at 1, 4 and 10 years post-infection, the 5 most frequent Vpu sequence variants observed at each time-point were studied, and showed variation in HLA-C downregulation between the viral quasi-species present at all timepoints (Fig 2E). Phylogenetic analysis has shown that in this individual a single virus from one timepoint gives rise to all of the quasispecies present at the next, indicating that in this individual persistent diversification in viral downregulation of HLA-C occurs (S2 Fig) [48]. HLA-C downregulation was characterized for 3 further donors where multiple quasi-species were tested at a single timepoint (Fig 2F–2H), and 2 further donors where dominant Vpu species were tested from longitudinal timepoints spanning 2 and 4 years (Fig 2I and 2J). Together this analysis of 41 viruses from 9 individuals shows robust changes in HLA-C downregulation in longitudinal infection are not readily observed, but that variation between quasi-species is detected in some individuals which could result in adaptation of downregulation in chronic infection.

### HLA-C downregulation adapts to host HLA genotype

**Fig 3:**
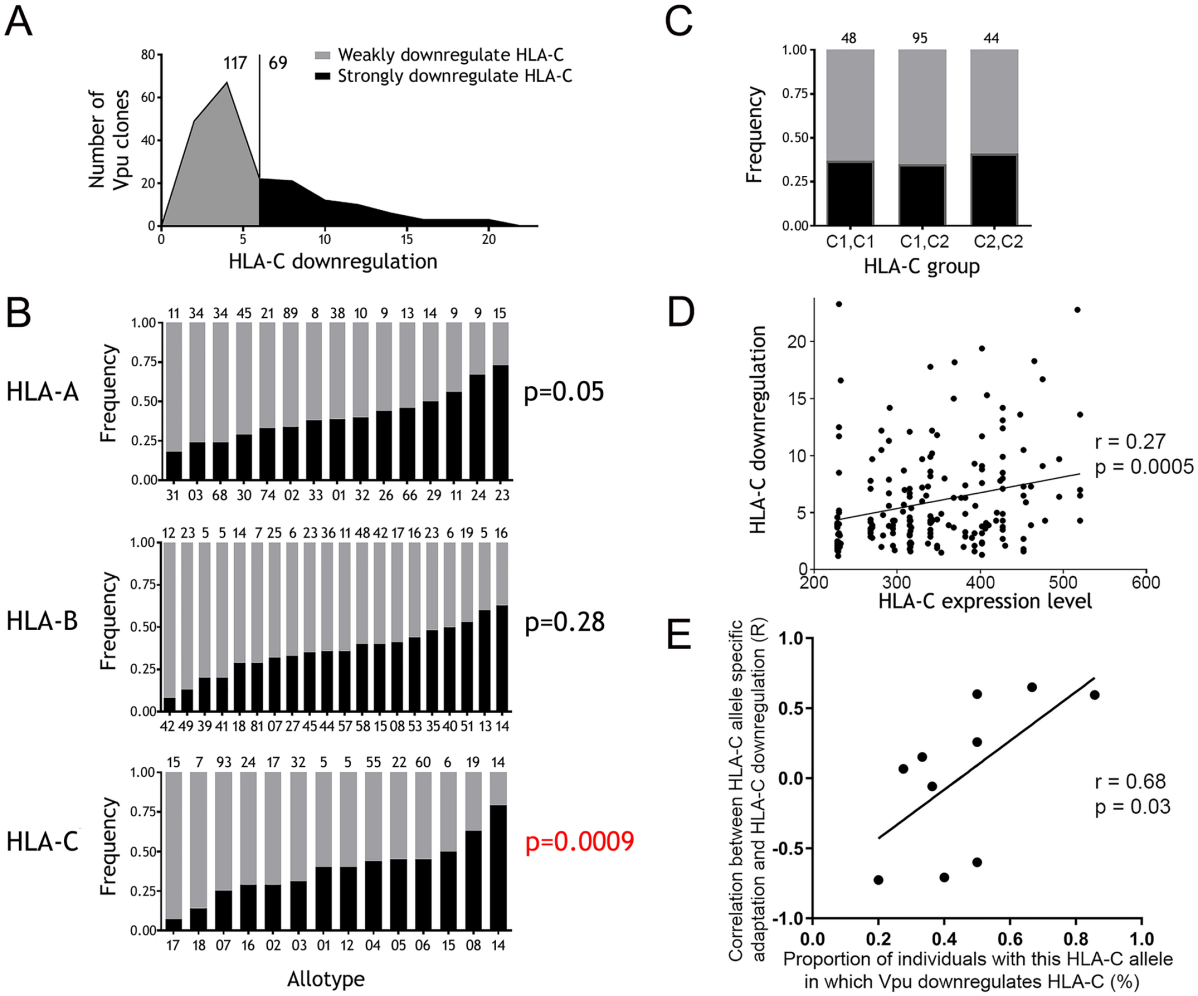
Downregulation of HLA-C shows adaptation to host HLA genotype. (A) For 186 individuals a single Vpu clone from chronic untreated infection was measured for ability to downregulate HLA-C by transfection of Molt-4 cells. The distribution of HLA-C downregulation is shown, with a threshold of 6-fold used to define 69 strongly downregulating (black) and 117 weakly downregulating Vpu clones (grey). (B) The proportion of Vpu clones that downregulate HLA-C strongly is shown when individuals are stratified by the presence of common alleles of each of the HLA-A,—B, and—C loci. The number of individuals in each group is shown above each plot and statistical analysis is by chi square tests. (C) HLA-C alleles can be grouped as C1 or C2 according to their differential binding of KIR2D receptors, and the proportion of Vpu clones that strongly downregulate HLA-C is shown for individuals when grouped by these genotypes. (D) HLA-C alleles differ in expression level and can be inferred for an individual from the HLA-C genotype. HLA-C expression level and the observed HLA-C downregulation by Vpu are plotted for 186 individuals. (E) For the subset of individuals where viral adaptation to host HLA-C alleles could be quantified (n = 72), the correlation between host HLA-C allele-specific viral sequence adaptation and observed downregulation of HLA-C by Vpu was determined. For 10 HLA-C alleles this correlation is plotted against the proportion of individuals with that HLA-C allele in which Vpu downregulates HLA-C. Correlations were determined using two-tailed Spearman analyses.

To identify the characteristics of infections in which HIV-1 differentially modulates HLA-C, we measured the ability of Vpu from 195 different infected individuals of the BC HOMER and UARTO cohorts to downregulate HLA-C. In all cases participants were ART naïve and in the chronic stage of infection, and HLA genotypes were known for 186 of the individuals. For each individual a single Vpu sequence was cloned from plasma, expressed by transfection in Molt-4 cells, and HLA-C downregulation quantified by flow cytometry. Vpu-mediated downregulation of HLA-C measured by transfection of Molt-4 cells, correlates robustly with HLA-C downregulation by primary HIV-1 viruses (S1 Fig). The observed distribution showed a natural threshold at approximately 6-fold downregulation of HLA-C, where in 117 of the infections Vpu downregulated HLA-C by <6-fold, and in 69 of the infections Vpu downregulated HLA-C >6-fold (Fig 3A). Subsequent categorical analyses used this threshold to classify Vpu molecules as strong, or weak downregulators of HLA-C. When individuals were stratified by common alleles of the classical HLA-I loci, the frequency of Vpu clones that downregulate HLA-C strongly varied significantly in association with host HLA-C allotype (p = 0.0009), but not with HLA-A (p = 0.05) or HLA-B (p = 0.28) (Fig 3B). At the extreme ends of this spectrum, only 1 out of 15 Vpu clones from individuals carrying HLA-C*17 was capable of downregulating HLA-C strongly, in contrast to 11 out of 14 Vpu clones from individuals carrying HLA-C*14. This suggests immune responses which drive the virus to downregulate HLA-C, vary in their frequency between individuals with different HLA-C alleles.

Inhibition of NK cells, mediated by KIR binding to HLA-C, may exert selection pressure on HLA-C expression. All HLA-C alleles resolve into two groups, C1 and C2, based upon a dimorphism at positions 77 and 80 which determines their binding to inhibitory two-domain KIR [40]. However, we found no difference in the frequency of Vpu clones that downregulated HLA-C strongly when individuals were grouped by presence of C1 versus C2 alleles (Fig 3C). This suggests that inhibition of NK cells may not be a significant factor in the selection pressures that influence Vpu-mediated downregulation of HLA. HLA-C alleles differ in their surface protein expression level on peripheral blood lymphocytes, enabling HLA-C expression level to be inferred for individuals on the basis of their HLA-C genotype [30,31]. For 186 Vpu molecules, the observed HLA-C downregulation showed a positive correlation with inferred HLA-C expression level in the host prior to infection (r = 0.27, p = 0.0005) (Fig 3D). This indicates that the higher the HLA-C expression level of an individual that is infected with HIV-1, the greater the downregulation of HLA-C demonstrated by the viral Vpu that adapts to this individual.

To maximize statistical power our primary analyses combined individuals from the BC HOMER and UARTO cohorts. To rule out confounding by the substantial differences between these cohorts, for example in ethnicity and sex ratio, analyses were repeated stratified by cohort. Fig 3B showed the frequency of Vpu clones that downregulate HLA-C strongly, varied in association with HLA-C allotype. This association was not significant in BC HOMER and UARTO participants separately (p = 0.053 and 0.23 respectively) but plots of alleles with n≥5 in both cohorts show these do typically have similar effects. For example HLA-C*14 has the highest frequency of Vpu clones that downregulate HLA-C strongly in both cohorts; HLA-C*04, 06 and 08 are intermediate; and HLA-C*03 and 07 are among the lowest in both cohorts S3A and S3B Fig). Fig 3D showed that the magnitude of HLA-C downregulation observed for a Vpu clone, correlated with HLA-C expression level prior to infection inferred from host HLA-C genotype. This correlation was significant in both BC HOMER (p = 0.008) and UARTO participants (p = 0.002) with highly similar correlation coefficients (S3C and S3D Fig). The total of 195 Vpu clones studied here also included viruses from the 4 major HIV-1 group M subtypes. Both viral ability to downregulate HLA-C and variation in this phenotype between primary viruses was observed within each subtype, consistent with prior study of 14 primary infectious molecular clones [26]. In this larger dataset we detect significant difference in the frequency of HLA-C downregulation between subtypes. Vpu clones from subtype A and B viruses more frequently downregulated HLA-C strongly than those from subtypes C or D (S4 Fig).

### Viral adaptation to HLA-C correlates with observed HLA-C downregulation

Given that the proportion of Vpu clones that downregulate HLA-C differs significantly based on host HLA-C genotype (Fig 3B), we hypothesized that that among individuals with an HLA-C allele that strongly selects for viral downregulation of HLA-C, there would be a direct correlation between the extent of viral adaptation to that HLA-C allele, and the observed HLA-C downregulation by Vpu. In contrast, such a relationship would not be observed in individuals carrying an HLA-C allele which does not select for viral downregulation of HLA-C. To assess this, we used a published HLA adaptation metric to quantify the extent of HIV-1 adaptation to host HLA-C alleles [49]. The strength of correlation between viral adaptation to host HLA-C and observed downregulation of HLA-C by Vpu was then determined for each HLA-C allele (S5 Fig). Consistent with our hypothesis, a significant positive correlation was observed between a given Vpu’s ability to downregulate HLA-C in individuals expressing HLA-C*08 (an allele that strongly selects for viral downregulation of HLA-C), but not in individuals expressing HLA-C*07 (an allele which does not select for viral downregulation of HLA-C). Across HLA-C alleles, the strength of these correlations associates directly with the variation between HLA-C alleles in selection pressure for HLA-C downregulation, shown by the frequency of Vpu clones that downregulate HLA-C in individuals with that HLA-C allele (r = 0.68, p = 0.03) (Fig 3E). Thus, the variation in HLA-C downregulation observed by viruses with different degrees of adaptation to an HLA-C allele, supports the finding that Vpu-mediated downregulation adapts to host HLA-C genotype.

### Variation in the Vpu transmembrane region determines downregulation of HLA-C

**Fig 4:**
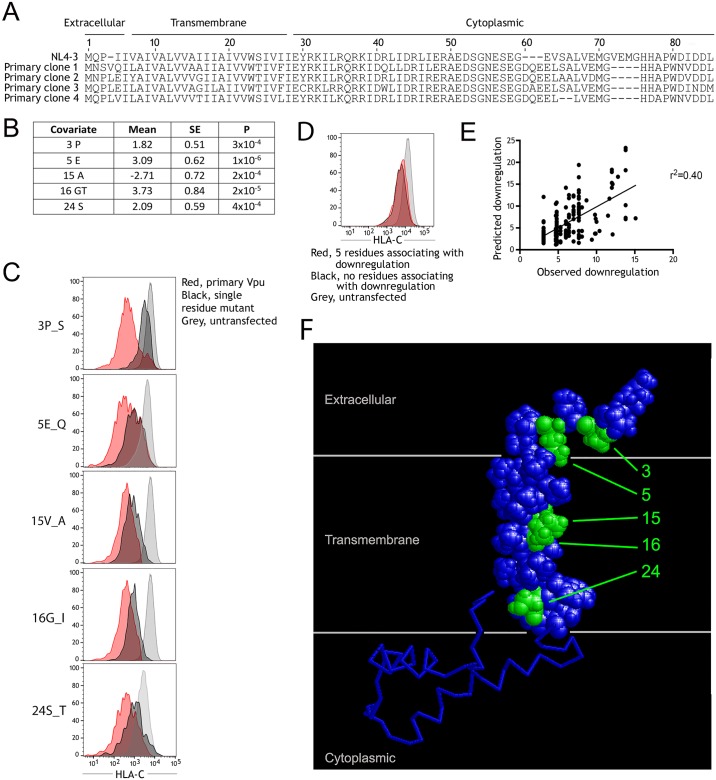
Variation in the Vpu transmembrane region is responsible for the range of HLA-C downregulation exhibited by primary HIV-1 viruses. (A) Sequence of representative Vpu clones that weakly (clone 1), or strongly downregulate HLA-C (clones 2–4), are shown aligned to Vpu from NL4-3. (B) A multiple linear regression model using 191 of the primary Vpu clones identifies 5 positions of Vpu at which the residues indicated associate independently with HLA-C downregulation. (C) These positions were each confirmed to influence HLA-C downregulation using single residue mutants of Vpu. HLA-C staining is shown for cells transfected with primary Vpu clones (red), single residue mutants (black), and untransfected cells (grey). (D) HLA-C staining for cells transfected with a Vpu constructed to have all 5 residues associated with HLA-C downregulation (red), or that with no residues associating with HLA-C downregulation (black), is shown compared to untransfected control (light grey). (E) HLA-C downregulation predicted by the model in panel B, compared to that observed for each of the 191 Vpu clones. (F) An NMR structure of Vpu, with N-terminal and transmembrane regions highlighted, shows the location of the 5 Vpu positions at which primary sequence variations associate with HLA-C downregulation (green).

For a small number of paired Vpu molecules that differentially downregulate HLA-C, sequence variants which explain the differences in HLA-C downregulation have been identified. For example, downregulation of HLA-C by Vpu from WITO but not NL4-3 is primarily explained by the sequence differences at positions 4 and 5 of Vpu [26]. However, these positions are not sufficient to predict HLA-C downregulation in natural Vpu sequences, indicating other residues also play a role. Using the nearly 200 Vpu clones from different individuals that were characterized for ability to downregulate HLA-C, we identified Vpu sequence variants which account for variation in HLA-C downregulation in this population of primary viruses. Vpu is an approximately 80 amino acid protein with a large cytoplasmic region, a transmembrane domain, and a short N-terminal stalk. Representative sequences of primary Vpu molecules that were observed to downregulate, or not to downregulate HLA-C, are shown aligned to Vpu from NL4-3 (Fig 4A). At each Vpu residue, amino acid polymorphism was tested for association with the observed downregulation of HLA-C for 191 Vpu clones. A multiple linear regression model identified 5 positions at which the residues indicated associated independently with HLA-C downregulation (Fig 4B). The strongest effects are presence of glutamic acid at position 5, or a glycine or threonine at position 16, which when present result in Vpu molecules which downregulate HLA-C more strongly. An alanine at position 15 associated with weaker downregulation of HLA-C. The frequency of different amino acids observed at each of these 5 positions is detailed in S6 Fig. These five positions remained independently significant when viral subtype is included as a covariate (S1 Table), and in analyses stratified by viral subtype or cohort each residue except for serine at position 24 remains significant in multiple subtypes and cohorts (S2 and S3 Tables).

Each of these 5 Vpu positions was confirmed to impact HLA-C downregulation, in the direction indicated, in the context of primary Vpu sequences compared to single amino acid mutants (Fig 4C). For example, a primary Vpu clone with proline at position 3 downregulated HLA-C strongly, but a mutant constructed with serine, the next most common residue at position 3, showed reduced ability to downregulate HLA-C. Although this population analysis identified 5 positions of importance, individual primary Vpu sequences carried no more than 3 of the residues detected to associate with HLA-C downregulation, in various combinations. When we generated a mutant Vpu with the variants that associate with greater HLA-C downregulation at all 5 of the positions implicated, this molecule demonstrated negligible downregulation of HLA-C, suggesting that some of these residues may not be compatible with one another (Fig 4D). The multiple linear regression model defined in Fig 4B, when used to predict HLA-C downregulation for each Vpu clone, explains 40% of the observed variation in HLA-C downregulation for these Vpu molecules (Fig 4E). A plot of the residuals between observed and predicted HLA-C downregulation confirms linear regression analysis is appropriate (S7 Fig). When highlighted on the NMR structure of Vpu, the 5 positions implicated in HLA-C downregulation cluster in the transmembrane and N-terminal regions of the molecule (Fig 4F) [50]. These 5 positions were also observed to vary in the longitudinal Vpu clones which showed variation in HLA-C downregulation (S2 Fig). Together these results suggest the transmembrane domain of Vpu is responsible for interaction with HLA-C, and that primary viruses exploit multiple alternative sequences which are able to downregulate HLA-C.

### The transmembrane domain of HLA mediates interaction with Vpu

**Fig 5:**
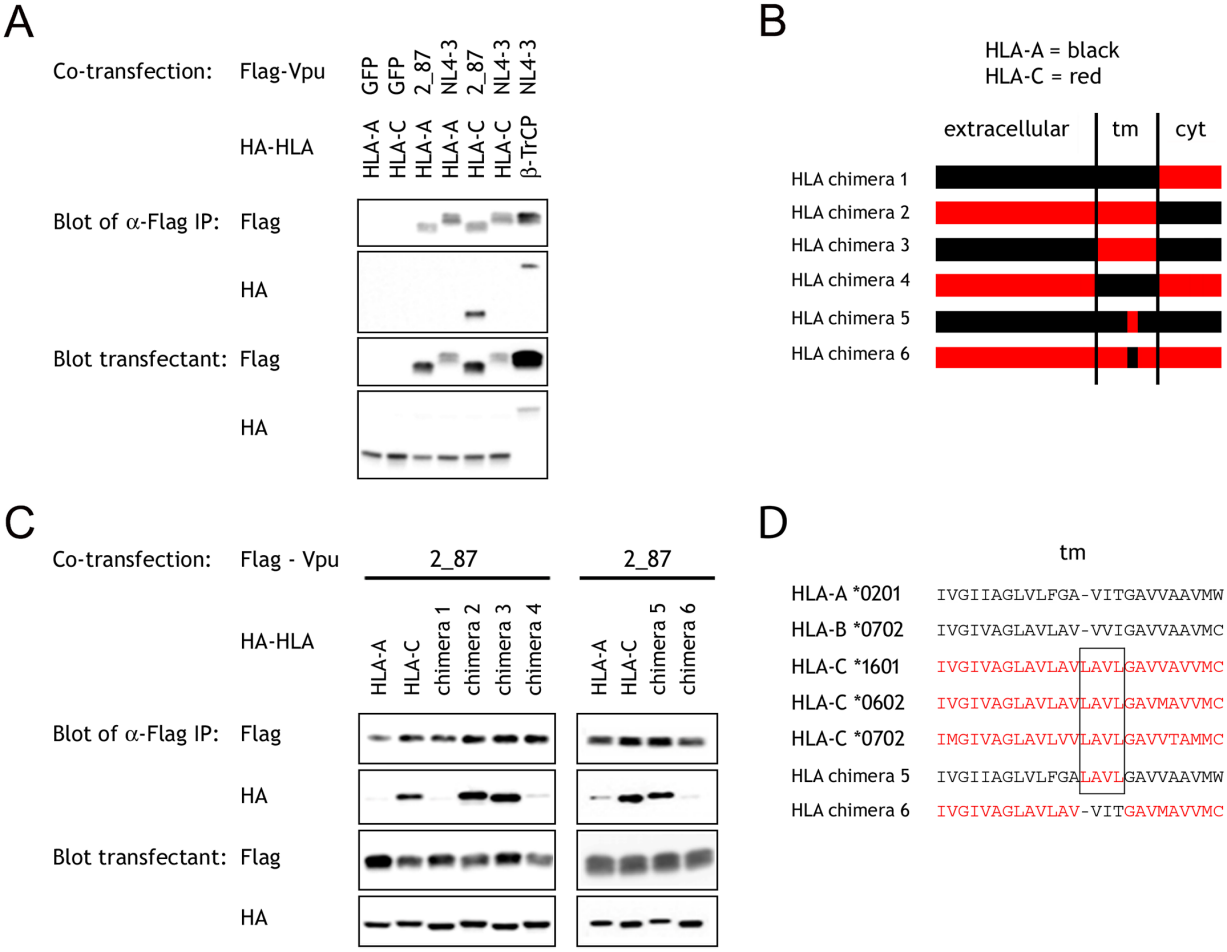
Transmembrane domain residues of HLA mediate the interaction with Vpu. (A) 293T cells were transfected with Flag-tagged constructs of Vpu from a primary virus 2_87, Vpu from NL4-3, or GFP as a negative control. Cells were co-transfected with HA-tagged constructs of HLA from the A or C loci, or HA-tagged β-TrCP as a positive control. Flag immunoprecipitation was followed by western blotting with anti-Flag or anti-HA antibody, and blots of the lysate prior to immunoprecipitation are also shown. (B) Chimeras 1–6 were constructed with the regions exchanged between HLA-A and HLA-C shown. (C) Co-immunoprecipitations were repeated using Vpu from 2_87 and the chimeric HLA constructs. (D) Sequences of the transmembrane domain of representative HLA-A and HLA-B alleles, 3 alleles which represent the transmembrane sequences of all common HLA-C alleles, and the chimeric constructs 5 and 6. Complete sequences for all HLA constructs are detailed in S8 Fig.

Analyses of HLA chimeras also indicate the transmembrane domain of HLA-C molecules is responsible for interaction with Vpu. HEK 293T cells were transfected with Flag-tagged constructs of Vpu from a primary virus, 2_87, which was previously shown to downregulate HLA-C, or from NL4-3 which does not downregulate HLA-C [26]. Cells were co-transfected with HA-tagged constructs of either HLA-A or HLA-C. Flag immunoprecipitation followed by western blotting with anti-Flag or anti-HA mAb, detected precipitated Vpu and associated HLA respectively. Vpu from 2_87 co-precipitated HLA-C, in contrast to lanes using Vpu from NL4-3 or the HLA-A molecule, showing an interaction between primary Vpu and HLA-C consistent with our cellular observations (Fig 5A). Experiments were repeated using chimeras between HLA-A and HLA-C, where the primary Vpu co-precipitated specifically chimeras 2 and 3 with transmembrane domains from HLA-C, but not chimeras 1 and 4 lacking HLA-C transmembrane sequence (Fig 5B and 5C). This indicates the transmembrane domain of HLA-C mediates interaction with Vpu. Alignment of HLA transmembrane domain sequences identified a 4 residue sequence, LAVL, which is conserved in HLA-C molecules which we have found downregulated by Vpu, but not in HLA-A/B which are unaffected by Vpu (Fig 5D) [26]. It was confirmed the primary Vpu could co-precipitate HLA chimera 5, comprised entirely of HLA-A with just these 4 transmembrane residues substituted from HLA-C, but not the reciprocal chimera 6 (Fig 5B–5D). Thus, studies of mutant HLA-I molecules implicate 4 residues in the transmembrane domain of HLA-C, that contribute to the interaction with Vpu.

## Discussion

Until recently it was believed that HIV-1 Nef subverted both CTL and NK cell immunity by downregulating the dominant CTL ligands HLA-A and -B, but preserving expression of HLA-C and HLA-E which serve as ligands for KIR2DL and NKG2A receptors that inhibit NK cells [16,18]. This understanding was modified by our finding that primary strains of HIV-1 can also downregulate HLA-C using the viral Vpu protein [26]. A major difference between the viral modulation of HLA-A/B compared to HLA-C, is that most HIV-1 strains downregulate HLA-A/B relatively well whereas HLA-C downregulation varies widely. The consequences resulting from HIV-1 downregulating HLA-C on infected cells in some individuals but not others are unclear. Variation between HIV-1 viruses provides an opportunity to identify selection pressures in human individuals which may be relevant. In this study we have characterized HLA-C downregulation for a wide range of primary viruses. We demonstrate that HIV-1 viruses from the latent reservoir of certain individuals downregulate HLA-C, that different quasispecies members can vary widely in their ability to downregulate HLA-C during chronic infection, and that viral downregulation of HLA-C shows a striking adaptation to the HLA genotype of the host.

In a screen of 128 replication competent viral isolates from limiting dilution outgrowth assays of 19 individuals treated with effective ART, 4 individuals harbored multiple viral isolates that downregulated HLA-C. These viruses are representative of the latent viral reservoir with the ability to seed new infection when ART is ceased [41]. Consequently, it will be necessary to clear reservoir viruses that are able to downregulate HLA-C to cure HIV-1 in certain individuals. This complicates some shock and kill strategies, which have proposed to use HLA-C restricted effectors as this HLA was previously thought to be preserved on infected cells [51,52]. It may also provide new opportunities for individualized therapy although such approaches, if ultimately developed, would be limited in scalability at least initially. Characterization of the CTL responses in the 4 individuals in which HIV-1 downregulates HLA-C may identify CTL epitopes that could represent candidates for expansion and treatment as immune therapies, due to their in vivo selection pressure suggested by this study. The unique role of HLA-C, exerting selection pressures that result in variable modulation of its expression, may make it possible for immunotherapies to achieve enhanced efficacy if both of the opposing pressures on HLA-C expression level could be established in the same individual.

In vitro infections of primary CD4+ T cells with transmitted/founder viruses, and virus from the same individual after 6 months of untreated infection, showed no change in HLA-C downregulation for 3 individuals. Analyses of 35 Vpu clones from 6 untreated individuals in chronic infection detected little longitudinal change in HLA-C downregulation, but confirmed variation between viral quasi-species present at a timepoint in several individuals, which could result in adaptation to host HLA genotype. These results indicate HLA-C downregulation by HIV-1 is not frequently affected by early innate immune pressures during acute infection [53,54]. A screen of Vpu molecules from nearly 200 chronically infected ART naïve individuals, found that in approximately one third of the infections viruses downregulated HLA-C strongly. Host HLA-C genotype was the strongest corollary of HLA-C downregulation identified. Viruses from individuals with HLA-C alleles such as C*07, generally downregulated HLA-C only weakly, whereas viruses from individuals with other alleles such as C*14 were far more likely to downregulate HLA-C strongly.

This differential downregulation of HLA-C is unlikely to be attributable to viral adaptation to the susceptibility of different HLA-C alleles to downregulation by Vpu. This is because primary HIV-1 viruses which downregulate HLA-C have been used to infect CD4+ cells from different individuals in vitro, and showed downregulation which was broadly similar across HLA-C types [26,27]. Further, the 4 HLA transmembrane residues found to be required for interaction with Vpu are conserved in all HLA-C alleles. We therefore interpret the association between downregulation and host HLA-C genotype to represent viral adaptation to differences in immune responses which are mediated by HLA-C alleles. It is well established that downregulation of HLA-A/B by Nef subverts CTL responses, so it seems likely that certain CTL responses are also the selection pressure resulting in HLA-C downregulation [18,19]. Consistent with this idea there is both in vitro and in vivo evidence that lower HLA-C expression levels can decrease the efficacy or frequency of HLA-C restricted CTL responses [26,31,32]. The variation in adaptation to HLA-C alleles that we observed, suggests that HLA-C alleles differ in their likelihood of restricting CTL responses which the virus responds to by downregulating HLA-C.

It is not clear what differentiates HLA-C alleles that are more likely to select for HLA-C downregulation, although they tend to be expressed at higher levels. We observed a direct correlation between the level of HLA-C expression by an individual prior to infection, and the degree of downregulation observed following chronic infection. Although certain HLA-C restricted CTL responses may be evaded by viral downregulation of HLA-C, in two thirds of infections Vpu downregulated HLA-C only weakly. Thus, there appears to be some advantage to the virus in preserving HLA-C expression in most individuals. Grouping HLA-C alleles based on differential KIR binding did not result in differences in the frequency of Vpu molecules that downregulate HLA-C strongly. This might be clarified by KIR typing but suggests that inhibition of NK cells may not be a significant factor in the selection pressures that influence viral downregulation of HLA-C, because the KIR bound by these groups of HLA-C alleles differ functionally, and these HLA-C allele groups do associate with other outcomes influenced by NK cells [40,55–57]. Other reasons why the virus might preserve HLA-C include a possible cost to replication efficiency of using Vpu to downregulate HLA-C. Vpu has multiple functions such as antagonizing CD4 and tetherin to promote virion release from infected cells, and these may be impaired when Vpu is used to downregulate HLA-C [58,59]. Interactions between different conformations of HLA-C and Env have also been reported to influence virion infectivity, and could represent another mechanism impacted by HLA-C downregulation [60]. That magnitude of downregulation is part of the viral adaptation to host HLA-C alleles, is supported by the variation observed in HLA-C downregulation by viruses with different degrees of adaptation to host HLA-C alleles.

Molecular characterization using primary virus derived Vpu sequences and chimeric HLA constructs identified 5 positions in the Vpu N-terminal stalk and transmembrane domain, and 4 residues in the HLA transmembrane domain, which are important in the interaction required for downregulation of HLA-C. The 5 positions of Vpu are all found on a similar face of the transmembrane/extracellular region of the molecule, consistent with residues at these sites promoting or disrupting a binding site. The 4 residues of the HLA transmembrane identified to be important are not present in any common HLA-A or—B alleles, but are conserved in all HLA-C alleles. The HLA-E transmembrane sequence differs from all classical HLA alleles in these 4 positions, so it will be interesting to determine if Vpu from different primary viruses can variably modulate HLA-E in addition to HLA-C [61]. Significantly, the Vpu variants identified are polymorphic in primary Vpu sequences, raising the possibility of predicting HLA-C downregulaton by primary virus from the Vpu sequence alone. This could identify preliminary characteristics of infections in which HIV-1 does or does not downregulate HLA-C for experimental validation, although additional effects of HIV-1 on HLA may occur in vivo. For example Vpu can suppress NF-kb activity to inhibit transcription from all HLA loci [62]. Variation between primary viruses in downregulation of HLA-C represents an opportunity to identify specific cellular immune responses that exert selection pressure on HLA expression in vivo. Comparison of cellular responses between individuals in which HIV-1 downregulates versus preserves HLA-C, has potential to identify specific CTL epitopes useful for HIV-1 therapy, and to improve understanding of the differential role of HLA-C molecules between infected individuals.

## Methods

### Ethics statement

Samples from HIV-1 infected individuals were used from the following existing collections. PBL from the Reservoir Characterization Support Section of the BELIEVE consortium, approved by the George Washington University Committee on Human Research (021750). Vpu and HLA genotypes from the BC HOMER and UARTO studies, approved by the Simon Fraser University Research ethics board (2016s0393) and the University of British Columbia-Providence Health Care Research ethics board (H11-01642 and H08-00962). All donors provided written informed consent and samples were de-identified for the present study.

### Detection and prediction of HLA-C expression

The mouse IgG2b monoclonal antibody DT9 was used in flow cytometry analyses to detect HLA-C [63]. This mAb has been shown to bind all alleles of HLA-C, but no common HLA-A/B alleles [30]. DT9 also recognizes HLA-E but this antigen is expressed at much lower levels than HLA-C on lymphocytes [2]. That the decreased DT9 binding of primary CD4+ cells upon HIV-1 infection reflects downregulation of HLA-C, has been confirmed by similar decreases in the staining of 3 different mAbs which recognize independent HLA-C epitopes but do not detect HLA-E [26]. Healthy individuals vary in their HLA-C expression level on PBL, and this has been shown to be a function of HLA-C genotype, with an average expression level for each allele defined (S4 Table). A sum of the average expression level of each HLA-C allele has previously been shown to predict HLA-C expression level for unrelated individuals of both European and African American ethnicity [31].

### HLA-C downregulation by inducible infectious HIV-1 reservoir isolates

QVOA were performed using a previously described protocol, with slight modifications [43]. Briefly, CD4+ T-cells from ART suppressed individuals were cultured at limiting dilutions, with 12 replicates per dilution, and stimulated with IL-2, PHA and irradiated allogeneic feeder cells from HIV-uninfected donors in the presence of Molt-4 cells [64]. Dilutions were chosen to minimize the probability that any given dilution will have all positive or all negative wells. After 14 days of culture, wells were screened for outgrowth of a viral isolate by ELISA for p24 (NCI Frederick). The following day, for the dilution with fewest CD4+ T cells that yielded wells with detectable virus, positive cultures were assayed by flow cytometry. After staining with mAb DT9 or an isotype control, detected by PE anti-mouse IgG (Sigma Aldrich), cells were blocked with murine IgG and further stained with CD3-BV421 (Sony Biotechnology) and Far Red Viability Dye (Thermofisher Scientific). Cells were then fixed and permeabilized using BD Cytofix/Cytoperm (BD Biosciences) followed by staining of intracellular Gag with KC57-FITC (Beckman Coulter). Staining was acquired using a SP6800 Spectral Cell Analyzer (Sony Biotechnology) and analyzed using FlowJo (Tree Star). Molt-4 cells lack CD3 expression, which enabled discrimination from primary T cells, before the median fluorescence intensity (MFI) of DT9 staining was compared between the HIV Gag+ (infected) and Gag- (uninfected) Molt-4 populations.

### HLA-C downregulation in primary cells infected with longitudinal viral clones

Paired infectious molecular clones from the transmitter/founder and 6-month timepoints of the same infected individual have been previously been described [46,47]. Single genome amplification from plasma RNA at these timepoints was followed by phylogenetic analysis of viral sequences to identify the transmitted virus and a biologically active 6 month consensus virus. Plasmids containing these infectious molecular clones were expressed in HEK 293T (ATCC, CRL-11268), used to infect primary CD4+ cells in vitro, and analyzed for HLA-C downregulation by flow cytometry all as previously described [26]. Briefly, CD4+ cells were magnetically selected, stimulated with anti-CD3/28 beads and IL-2 for 3–5 days, infected with HIV-1 and cultured for a further 6 or 7 days. Cells were then incubated with mAb DT9 or isotype control, followed by PE-anti-mouse IgG (Sigma-Aldrich). Free secondary antibody- binding sites were blocked with murine immunoglobulin before further staining with CD4-PB (BioLegend), CD8-APC and CD3-APC.Cy7 (both from Becton Dickinson), and yellow fluorescent reactive viability dye (Invitrogen). Cells were then fixed and permeabilized by incubation with paraformaldehyde and saponin (Becton Dickinson) before staining of HIV-1 intracellular Gag with KC57-FITC (Beckman Coulter). CD4 lymphocytes were discriminated as CD3+, viability dye and CD8-, and the MFI of DT9 staining was compared between Gag+ and Gag- populations.

### Vpu genes cloned from primary viruses

Plasma-derived Vpu clones in a Rev-dependent CRV1 based vector have previously been described for 14 longitudinally sampled, untreated, subtype B HIV-1 infections [48]. For 2 individuals from that study, long term non-progressors 1 and 5, multiple Vpu clones were characterized here for ability to downregulate HLA-C (clones 1–15 and 26–28 respectively). For 4 untreated subtype C infected individuals (CH596, CH492, CH256, CH694), for which plasma sequencing has previously been described [53,65,66], Vpu clones were constructed in the same vector and tested for HLA-C downregulation (clones 16–19, 20–25, 29–30 and 31–35 respectively). Sequences of these Vpu clones are given in S5 Table. Chronically infected ART-naïve individuals have previously been described from the BC HOMER cohort with subtype B infection [67,68] and the UARTO cohort with infections of subtypes A, C and D [69]. Overall, 25% of BC HOMER cohort participants reported they were men who have sex with men, while 39% reported a history of injection drug use, and 92% of the BC HOMER participants in this present study were male. 30% of the UARTO cohort samples included in this study were male and this cohort comprises predominantly heterosexual individuals. To characterize HLA-C downregulation a single plasma-derived Vpu sequence for 195 of these individuals, was cloned into a bicistronic pSel_RRE_–Vpu vector that expresses Vpu and GFP [70]. Of these 195 clones, Vpu sequences were of subtypes A (n = 45), B (n = 75), C (n = 19) or D (n = 56). Sequences of these Vpu clones are given in S6 Table. A panel of 14 infectious molecular clones of HIV-1, including viruses of subtypes B, C and D, have previously been characterized for ability to downregulate HLA-C when infecting primary CD4+ cells in vitro [26]. Vpu genes from each of these viruses were also generated in the pSel_RRE_–Vpu expression vector. Synthetic DNA sequences containing the desired Vpu sequence (Synthetic Genomics Inc) were inserted into the plasmid digested with AscI and SacII using Gibson Assembly (New England Biolabs), and chemically competent cells transformed according to manufacturer instructions. Transformed cells were selected on zeocin agar plates (Invivogen), single colonies grown in TB with Zeocin (Invivogen), plasmid extracted, and sequence verified using primer 5’-ACCTTGTTTATTGCAGCTT-3’ for Sanger sequencing (Integrated DNA Technologies).

### HLA-C downregulation by transfected Vpu clones

HeLa cells (ATCC, CCL-2) were grown in RPMI 1640 with 10% FBS, penicillin-streptomycin, and L-glutamine. Molt-4 cells were grown in the same media supplemented with 10mM HEPES. The 35 Vpu clones obtained in CRV1 based vectors, representing multiple quasi-species and longitudinal samples from 6 individuals, were assessed for ability to downregulate HLA-C in HeLa cells as previously described [26]. Briefly, TransIT-HeLaMONSTER (Mirus) was used to co-transfect HeLa with the CRV1 based vector containing Rev and primary Vpu genes, and with an EGFP expressing plasmid [48]. After 24hrs trypsinized cells were incubated with mAb DT9 or isotype control, followed by APC-conjugated anti-mouse IgG (BioLegend), and staining results were acquired using a FACSCalibur flow cytometer (Becton Dickinson). The 195 Vpu clones derived in pSel_RRE_–Vpu vectors, from chronically infected UARTO and BC HOMER cohort individuals, were assessed for ability to downregulate HLA-C in Molt-4 cells. Lipofectamine (ThermoFisher) was used according to manufacturer instructions to co-transfect ~200,000 cells with the pSel_RRE_–Vpu plasmid containing GFP and primary Vpu genes, and with the Rev expression plasmid pSel-Rev-ΔGFP previously described [70]. After 24hrs cells were stained with DT9 and an APC-conjugated secondary as above, and staining results were acquired using a SP6800 Spectral Cell Analyzer (Sony Biotechnology). 14 Vpu genes from HIV-1 infectious molecular clones were characterized for HLA-C downregulation in both HeLa cells transfected using the Mirus reagent, and Molt-4 cells transfected using Lipofectamine, as described above. All cytometry analysis was performed using FlowJo (Tree Star). Vpu transfected cells were discriminated from untransfected cells by GFP fluorescence, and the MFI of DT9 staining compared between these populations to calculate fold downregulation of HLA-C.

### Analysis of viral adaptation to host HLA-C genotype

Extent of viral sequence adaptation to host HLA-C genotype was determined for 72 subtype B-infected individuals from the BC HOMER cohort for whom HLA-I genotypes were available, using a published adaptation metric [49]. Briefly, a probabilistic model is used to compare the expected HIV-1 sequence if evolving indefinitely in a host whose immune system targeted solely viral epitopes restricted by the HLA allele in question, compared to if the virus was evolving indefinitely in the absence of immune pressure. The resulting ratio of these two scenarios is scaled so that it ranges from −1 to +1, where the extremes denote zero and complete adaptation to the HLA allele in question, respectively. This quantification of adaptation used specifically the viral Nef sequence because of the high number of HLA-associated sequence variants contained therein, and was restricted to HIV-1 subtype B as HLA-associated polymorphisms are extensively mapped for this subtype [38].

### Identification of Vpu residues influencing HLA-C downregulation

Of the 195 Vpu clones from individuals chronically infected with HIV-1 that were characterized for ability to downregulate HLA-C, 4 were excluded due to substantial sequence length polymorphism. The remaining 191 Vpu sequences were aligned and at every position of Vpu, polymorphic variants were tested for association with HLA-C downregulation as a categorical variable using Fisher’s exact test. Residues that differed with p<0.05 were then tested in a multiple linear regression analysis, identifying 5 positions with independently significant effects on downregulation of HLA-C. These were confirmed experimentally using single amino acid mutants of primary Vpu sequences generated by incorporating synthetic DNA sequences into the pSel_RRE_–Vpu vector, as described above for the construction of Vpu genes from 14 infectious molecular clones. HLA-C downregulation by mutant and primary Vpu clones was compared by transfection of Molt-4 cells, also as described above.

### Co-precipitation of chimeric HLA molecules with Vpu

A nine–amino acid HA epitope tag (YPYDVPDYA) was added to the N-terminus of HLA-A*0201, HLA-C*0501, or 6 chimeras of these HLA. The HA tag was added to the mature protein, by insertion just after the leader sequence, with addition of a second glycine-serine N-terminal to the tag to reproduce the cleavage site of the HLA-I molecule as previously described [71]. These constructs were synthesized by IDT with 5’ EcoRI and kozak sequences, and 3’ NotI recognition site, and inserted by Gibson Assembly into a pcDNA3.1(+) vector digested with EcoRI and NotI (all New England Biolabs). Transformed cells were selected with Ampicillin and clones sequenced with CMV3'F 5’-GGTAGGCGTGTACGGTGGGA-3’ and BGH rev 5’-TAGAAGGCACAGTCGAGG-3’. Immunoprecipitation of Vpu and detection of co-precipitated β-TrCP has been previously described [72]. This was repeated with an adapted method substituting HLA for β-TrCP. Briefly, HEK 293T cells were co-transfected with the HA-tagged HLA plasmid and Flag-tagged Vpu. 24 hours after transfection, cells were lysed in buffer containing 50mM Tris-HCl pH7.5, 100mM NaCl, 1mM EDTA, 2mM DTT, 0.1% NP40 and complete protease inhibitors (Roche), immunoprecipitated with rabbit anti-Flag monoclonal antibody (Sigma) and protein G agarose beads, then analyzed by western blot using mouse anti-Flag or anti-HA antibodies.

### Statistical analyses

Differences in HLA-C downregulation between reservoir isolates were compared by unpaired t tests. Variation in the frequency of Vpu clones that downregulate HLA-C strongly, between individuals of different HLA genotypes, was analyzed by chi square tests. Linear regression with Spearman analysis was used to determine correlations between observed HLA-C downregulation and predicted HLA-C expression level for different individuals, and across HLA-C alleles in terms of the frequency of Vpu clones that downregulate HLA-C, and the strength of correlation between HLA-C allele specific adaptation and HLA-C downregulation. Multiple linear regression models were used to analyze Vpu positions associating with downregulation of HLA-C. Significance was based on a two-sided p value ≤0.05 throughout. All analyses were performed in GraphPad Prism 6 or R version 3.4.3 statistical software packages [73].

## Supporting information

S1 Fig HLA-C downregulation quantified for 14 primary viruses, comparing transfection of cloned Vpu molecules to in vitro infection of primary cells. (A) HeLa cells were transfected with Vpu cloned from NL4-3 or a primary virus that downregulates HLA-C. HeLa populations were then gated based on the expression of GFP, and the MFI of HLA-C staining compared between GFP+ and GFP- cells from the same well. (B) Representative staining demonstrating the Vpu clone from NL4-3 does not down-regulate HLA-C, whereas the Vpu clone from the primary virus strongly downregulates HLA-C. (C) Comparison of HLA-C downregulation measured for 14 primary viruses, when infecting primary CD4+ cells in vitro (x-axis) [26] or cloned Vpu molecules are assayed by transfection (y-axis). (D-F) Replicate experiments using transfection of Molt-4 cells.
(TIF)

S2 Fig Vpu sequence information for the longitudinally sampled individual studied in Fig 2E. (A) A phylogenetic tree of 87 Vpu sequences previously obtained from this individual [48]. Sequences are shown from samples taken 1 (light blue), 4 (dark blue), or 10 (magenta) years after seroconversion. The tree is rooted using NL4.3 and a consensus subtype B sequence (black), and Vpu sequences tested for HLA-C downregulation are highlighted (boxed red). (B) For the 15 Vpu molecules that were tested for HLA-C downregulation in Fig 2E, the N-terminal sequences are shown. Vpu positions that were identified to contribute to HLA-C downregulation in the analysis of Fig 4 are highlighted, with green and red marking residues that associated with stronger and weaker downregulation of HLA-C respectively.
(TIF)

S3 Fig Stratification by cohort of analyses indicating HLA-C downregulation adapts to host HLA genotype. (A,B) Separately for BC HOMER and UARTO individuals, the proportion of Vpu clones that downregulate HLA-C strongly is shown for HLA-C alleles with n≥5 in both cohorts. The number of individuals in each group is shown above each plot. (C,D) HLA-C expression level for an individual inferred from HLA-C genotype, and observed HLA-C downregulation for Vpu from that individual, are plotted separately for BC HOMER and UARTO individuals. Correlations were determined Spearman analyses.
(TIF)

S4 Fig HIV-1 subtypes differ in their frequency of HLA-C downregulation. Data is shown for 195 individuals from which HLA-C downregulation by a single Vpu clone from chronic untreated infection was measured by transfection of Molt-4 cells. HLA-C downregulation is observed more frequently for Vpu molecules from viral subtypes A and B, compared to subtypes C and D, when analyzed by Fisher’s exact test.
(TIF)

S5 Fig Correlations between viral sequence adaptation to host HLA-C and the observed downregulation of HLA-C by Vpu. For 72 individuals with subtype B virus the extent of HIV-1 adaptation to each HLA-C could be quantified from the proportion of Nef sequence variants which associate with that HLA-C allele, which are observed in individual viral sequences. The strength of correlation between this viral adaptation to host HLA-C, and the observed downregulation of HLA-C by Vpu, was then determined for each HLA-C allele. (A) Representative linear regression analyses for C*07 where in 29 individuals sequence adaptation does not correlate with downregulation, or for C*08 where in 9 individuals sequence adaptation does correlate with downregulation of HLA-C by Vpu. (B) For each HLA-C allele is shown the number of individuals analyzed (n), the observed correlation between adaptation and downregulation (R), and among the individuals included in this analysis the fraction of the individuals with that allele for which Vpu demonstrated greater than 6-fold downregulation of HLA-C in transfected Molt-4 cells.
(TIF)

S6 Fig Vpu sequence variation observed at positions identified as important in downregulation of HLA-C. 191 primary Vpu clones were analyzed in Fig 4 and identified residues at Vpu positions 3, 5, 15, 16 and 24 which associated independently with HLA-C downregulation. For each of these positions the frequency of all residues observed in this population of 191 primary Vpu sequences is shown, for Vpu molecules found to downregulate HLA-C (black) and those that do not (grey).
(TIF)

S7 Fig Residuals plot for predicted HLA-C downregulation. In Fig 4E a multiple linear regression model was used to predict downregulation of HLA-C based on Vpu sequence at 5 positions. The residuals between observed and predicted HLA-C downregulation are shown for all 191 Vpu clones in this analysis.
(TIF)

S8 Fig Sequences of HA-tagged HLA constructs. Black text indicates sequence from HLA-A, red indicates sequence from HLA-C, with green identifying the HA tag added at the N-terminus after the leader peptide.
(TIF)

S1 Table Multiple linear regression model including virus subtype as a covariate. Using 191 primary Vpu clones from chronic infection a multiple linear regression model identified residues at 5 positions of Vpu affecting HLA-C downregulation (Fig 4B, shown on the left). This analysis was repeated including viral subtype as a covariate (subtype A, B, C, or D) and all positions remain independently significant, shown on the right.
(DOCX)

S2 Table Multiple linear regression model for viruses of each subtype. Using 191 primary Vpu clones from chronic infection a multiple linear regression model identified residues at 5 positions of Vpu affecting HLA-C downregulation (Fig 4B, shown on the left). This analysis was repeated separately for infections with viruses of different subtype (shown right). Blacked box indicates n = 0.
(DOCX)

S3 Table Multiple linear regression model for viruses of each cohort. Using 191 primary Vpu clones from chronic infection a multiple linear regression model identified residues at 5 positions of Vpu affecting HLA-C downregulation (Fig 4B, shown on the left). This analysis was repeated separately for individuals of each of the two cohorts used (shown right).
(DOCX)

S4 Table Average expression level of HLA-C alleles. The average expression level for each 2-digit HLA-C allele is reported here, from previously described staining of peripheral blood CD3+ cells from healthy donors analyzed by flow cytometry using the monoclonal antibody DT9 [31]. MFI of DT9 staining was plotted twice for each donor, once for each 2-digit HLA-C allele present, and the average MFI for each HLA-C allele is shown with N indicating the number of observations from which each average was determined.
(DOCX)

S5 Table Amino acid sequences of the 35 Vpu clones analyzed from 6 individuals in Fig 2. (DOCX)

S6 Table Amino acid sequences of the single Vpu clones analyzed for 195 individuals in Figs 3 and 4 and S3 Fig. (DOCX)
