# Supplementary figures and images for: HLA-C downregulation by HIV-1 adapts to host HLA genotype
Source: PLoS Pathog. 2018 Sep 4;14(9):e1007257. doi: 10.1371/journal.ppat.1007257 (PMC6138419; doi:10.1371/journal.ppat.1007257)

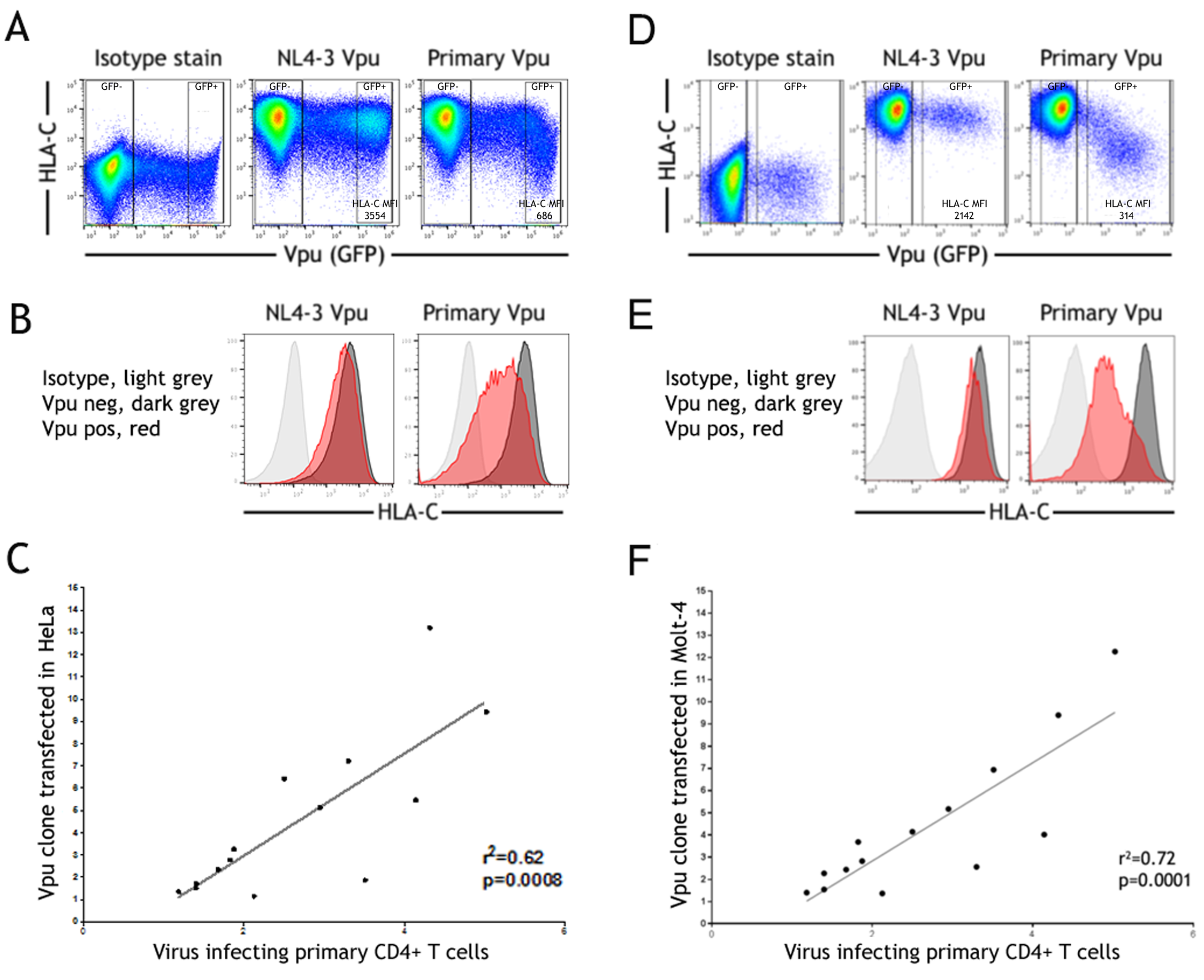

Supplement: S1 Fig — (A) HeLa cells were transfected with Vpu cloned from NL4-3 or a primary virus that downregulates HLA-C. HeLa populations were then gated based on the expression of GFP, and the MFI of HLA-C staining compared between GFP+ and GFP- cells from the same well. (B) Representative staining demonstrating the Vpu clone from NL4-3 does not down-regulate HLA-C, whereas the Vpu clone from the primary virus strongly downregulates HLA-C. (C) Comparison of HLA-C downregulation measured for 14 primary viruses, when infecting primary CD4+ cells in vitro (x-axis) [26] or cloned Vpu molecules are assayed by transfection (y-axis). (D-F) Replicate experiments using transfection of Molt-4 cells. (TIF) [file ppat.1007257.s001.tif]

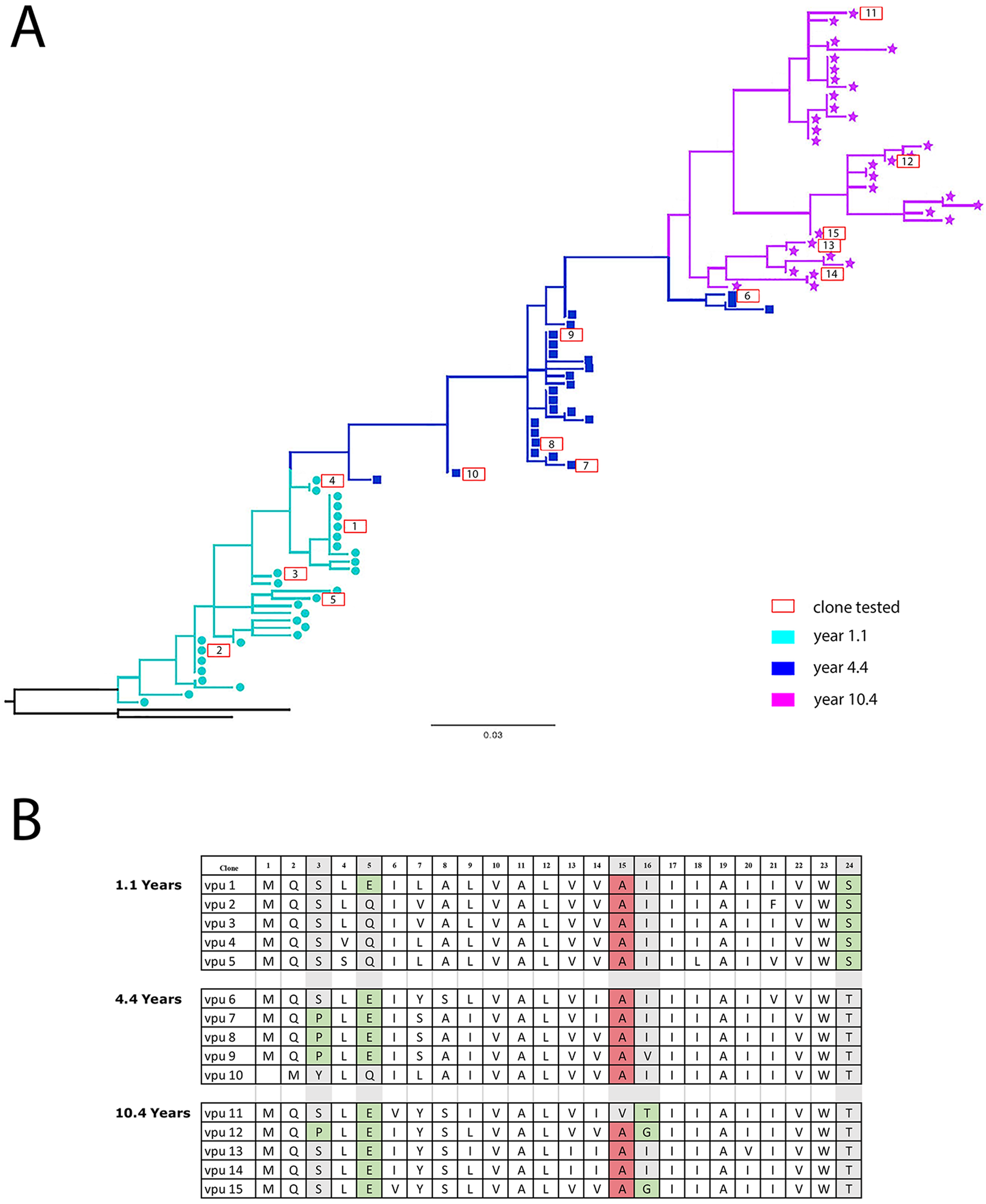

Supplement: S2 Fig — (A) A phylogenetic tree of 87 Vpu sequences previously obtained from this individual [48]. Sequences are shown from samples taken 1 (light blue), 4 (dark blue), or 10 (magenta) years after seroconversion. The tree is rooted using NL4.3 and a consensus subtype B sequence (black), and Vpu sequences tested for HLA-C downregulation are highlighted (boxed red). (B) For the 15 Vpu molecules that were tested for HLA-C downregulation in Fig 2E, the N-terminal sequences are shown. Vpu positions that were identified to contribute to HLA-C downregulation in the analysis of Fig 4 are highlighted, with green and red marking residues that associated with stronger and weaker downregulation of HLA-C respectively. (TIF) [file ppat.1007257.s002.tif]

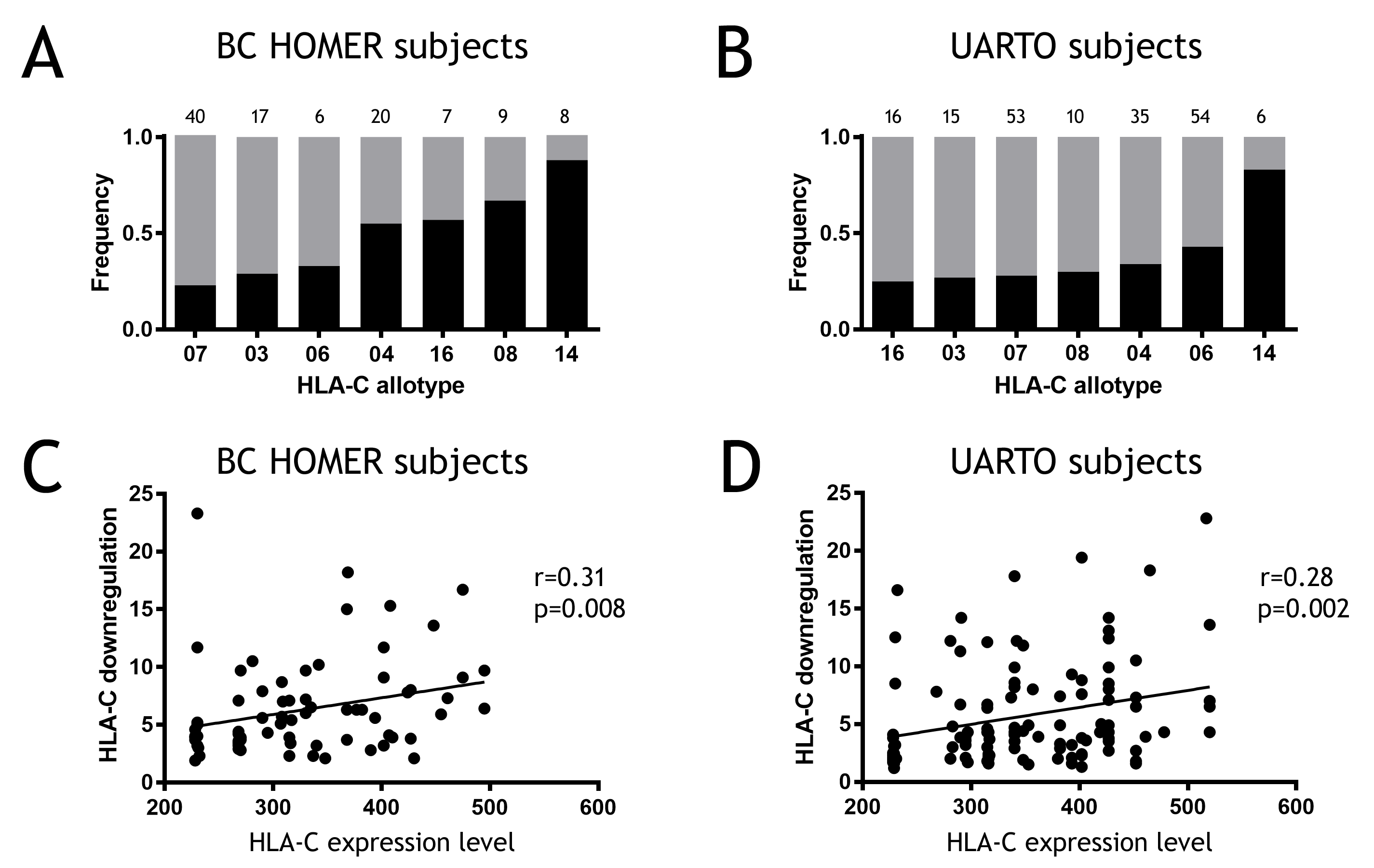

Supplement: S3 Fig — (A,B) Separately for BC HOMER and UARTO individuals, the proportion of Vpu clones that downregulate HLA-C strongly is shown for HLA-C alleles with n≥5 in both cohorts. The number of individuals in each group is shown above each plot. (C,D) HLA-C expression level for an individual inferred from HLA-C genotype, and observed HLA-C downregulation for Vpu from that individual, are plotted separately for BC HOMER and UARTO individuals. Correlations were determined Spearman analyses. (TIF) [file ppat.1007257.s003.tif]

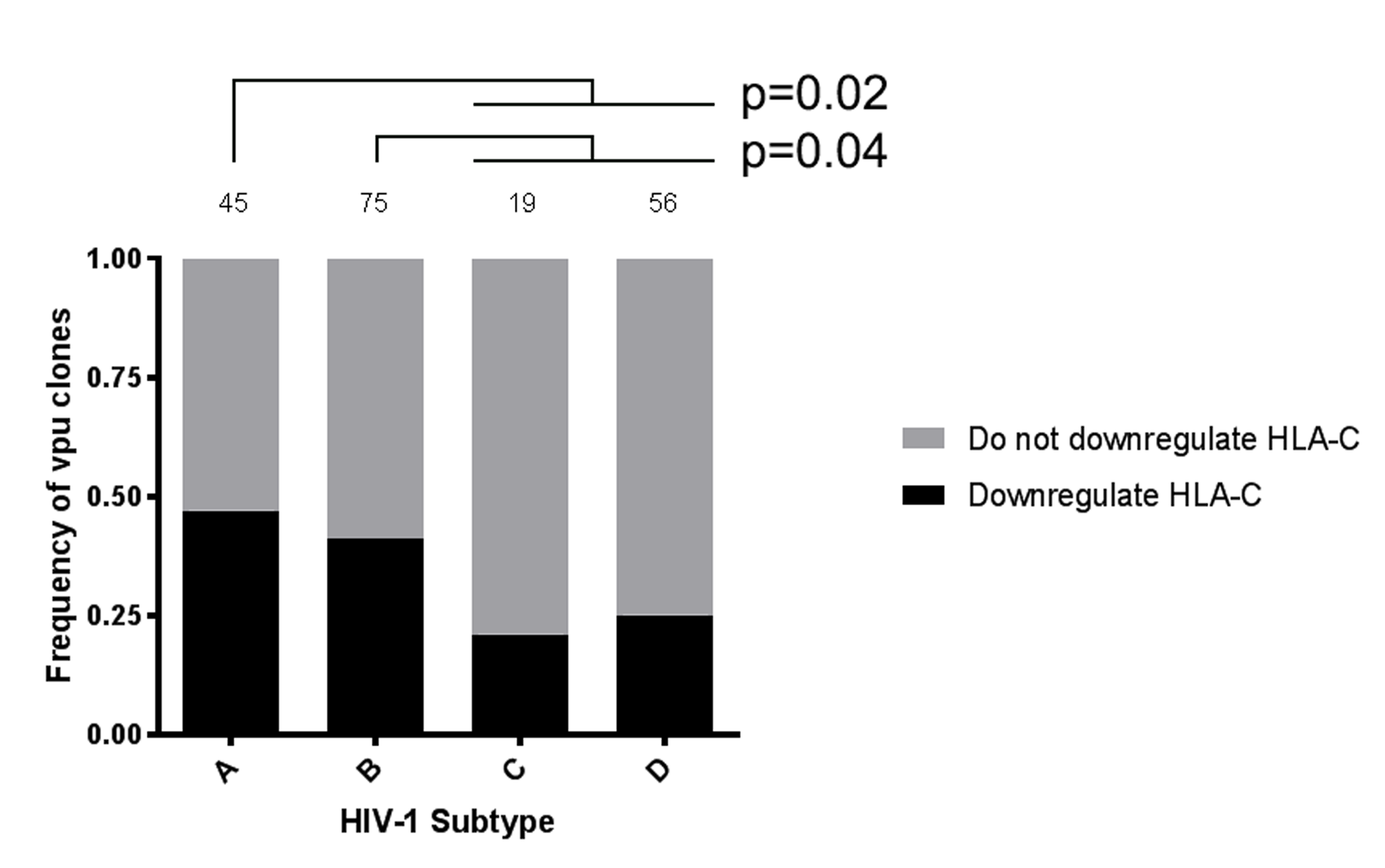

Supplement: S4 Fig — Data is shown for 195 individuals from which HLA-C downregulation by a single Vpu clone from chronic untreated infection was measured by transfection of Molt-4 cells. HLA-C downregulation is observed more frequently for Vpu molecules from viral subtypes A and B, compared to subtypes C and D, when analyzed by Fisher’s exact test. (TIF) [file ppat.1007257.s004.tif]

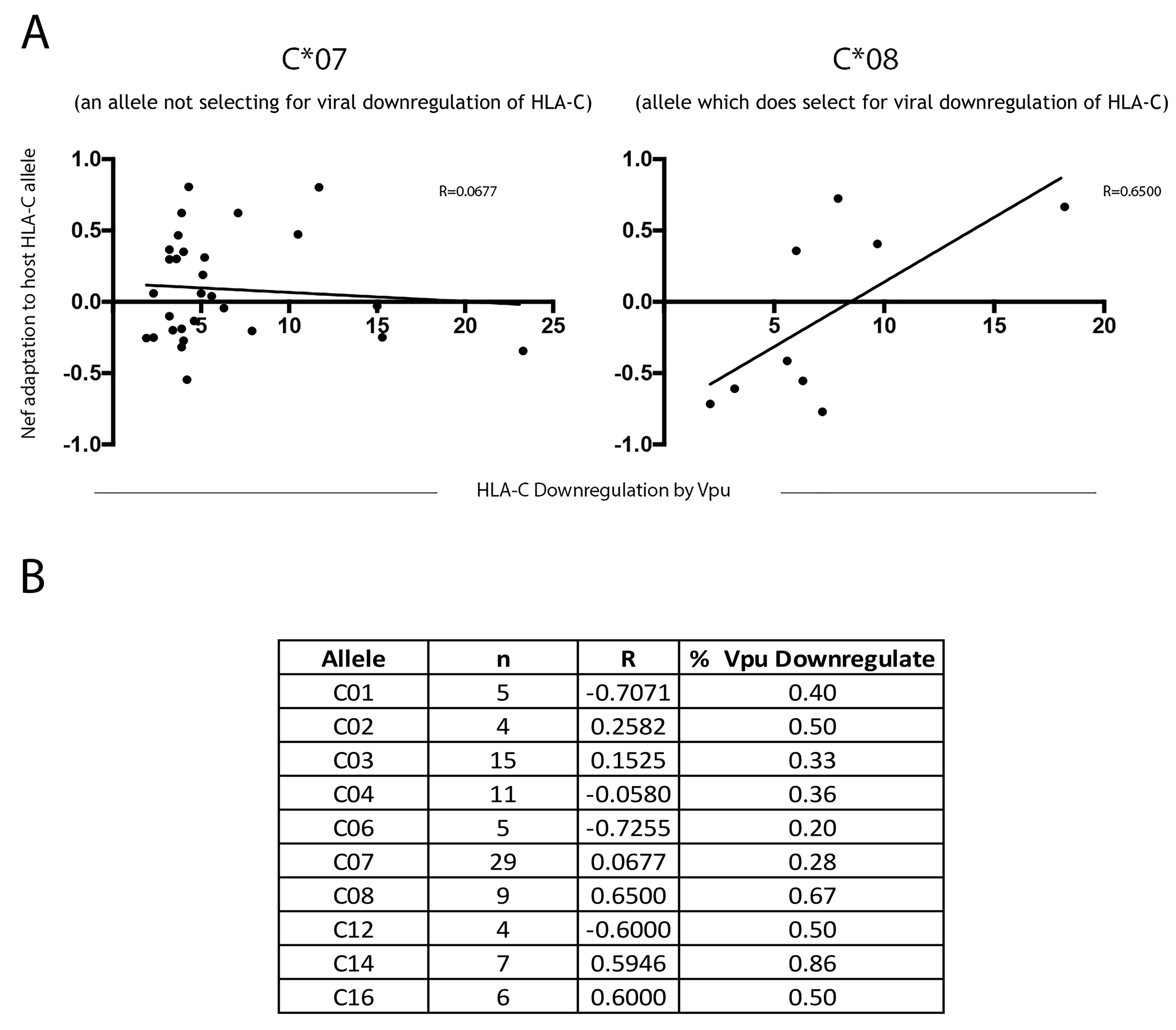

Supplement: S5 Fig — For 72 individuals with subtype B virus the extent of HIV-1 adaptation to each HLA-C could be quantified from the proportion of Nef sequence variants which associate with that HLA-C allele, which are observed in individual viral sequences. The strength of correlation between this viral adaptation to host HLA-C, and the observed downregulation of HLA-C by Vpu, was then determined for each HLA-C allele. (A) Representative linear regression analyses for C*07 where in 29 individuals sequence adaptation does not correlate with downregulation, or for C*08 where in 9 individuals sequence adaptation does correlate with downregulation of HLA-C by Vpu. (B) For each HLA-C allele is shown the number of individuals analyzed (n), the observed correlation between adaptation and downregulation (R), and among the individuals included in this analysis the fraction of the individuals with that allele for which Vpu demonstrated greater than 6-fold downregulation of HLA-C in transfected Molt-4 cells. (TIF) [file ppat.1007257.s005.tif]

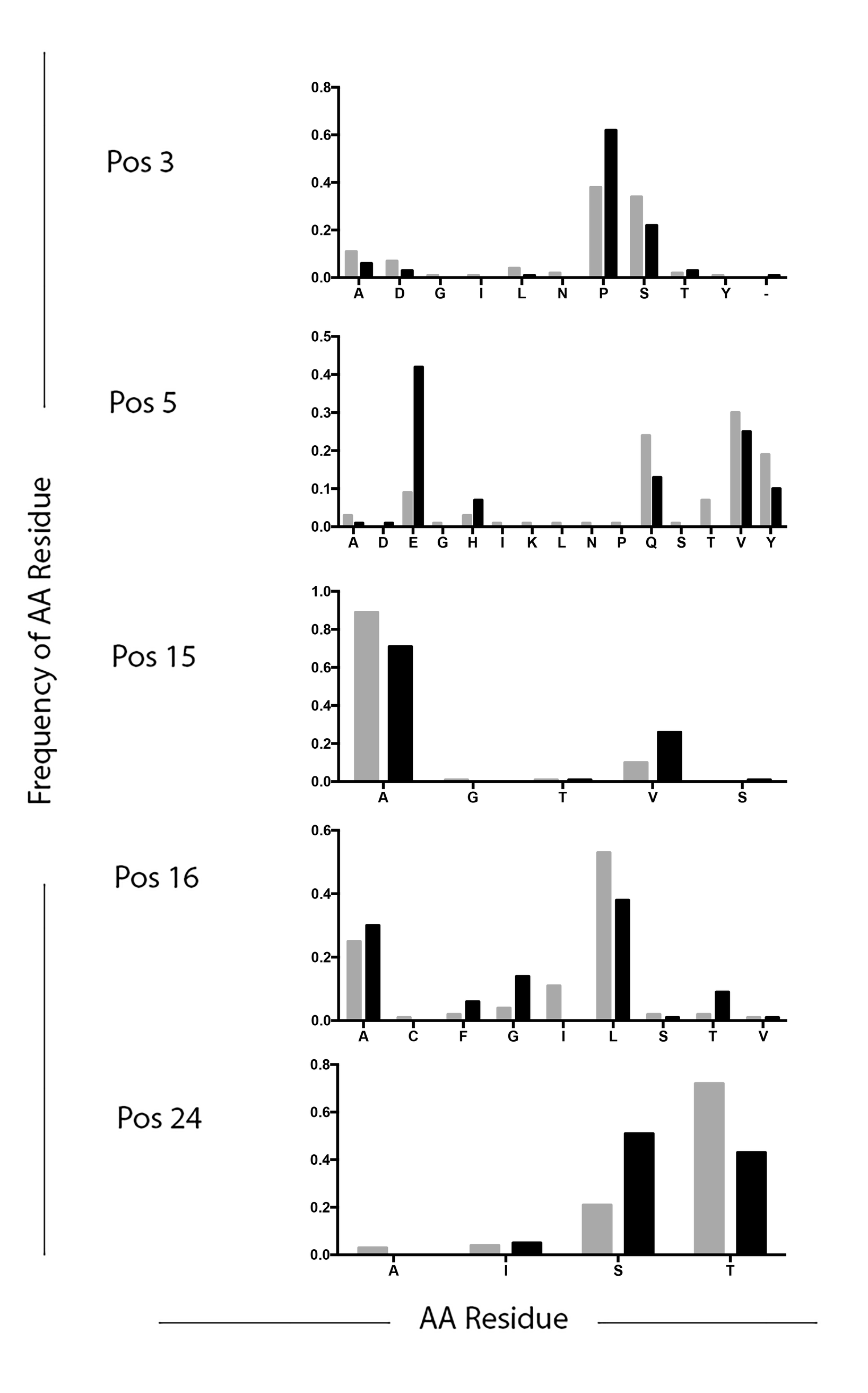

Supplement: S6 Fig — 191 primary Vpu clones were analyzed in Fig 4 and identified residues at Vpu positions 3, 5, 15, 16 and 24 which associated independently with HLA-C downregulation. For each of these positions the frequency of all residues observed in this population of 191 primary Vpu sequences is shown, for Vpu molecules found to downregulate HLA-C (black) and those that do not (grey). (TIF) [file ppat.1007257.s006.tif]

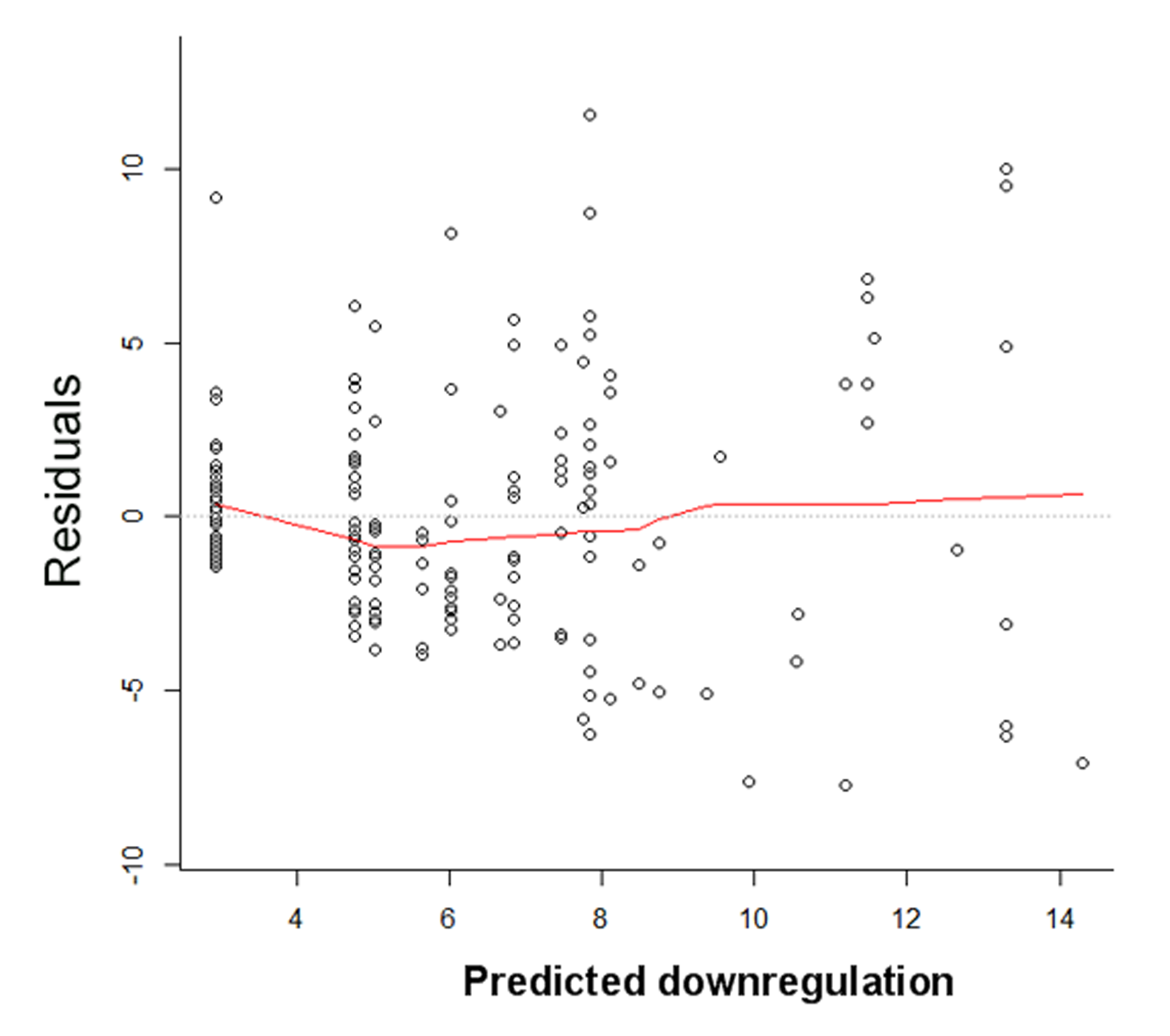

Supplement: S7 Fig — In Fig 4E a multiple linear regression model was used to predict downregulation of HLA-C based on Vpu sequence at 5 positions. The residuals between observed and predicted HLA-C downregulation are shown for all 191 Vpu clones in this analysis. (TIF) [file ppat.1007257.s007.tif]

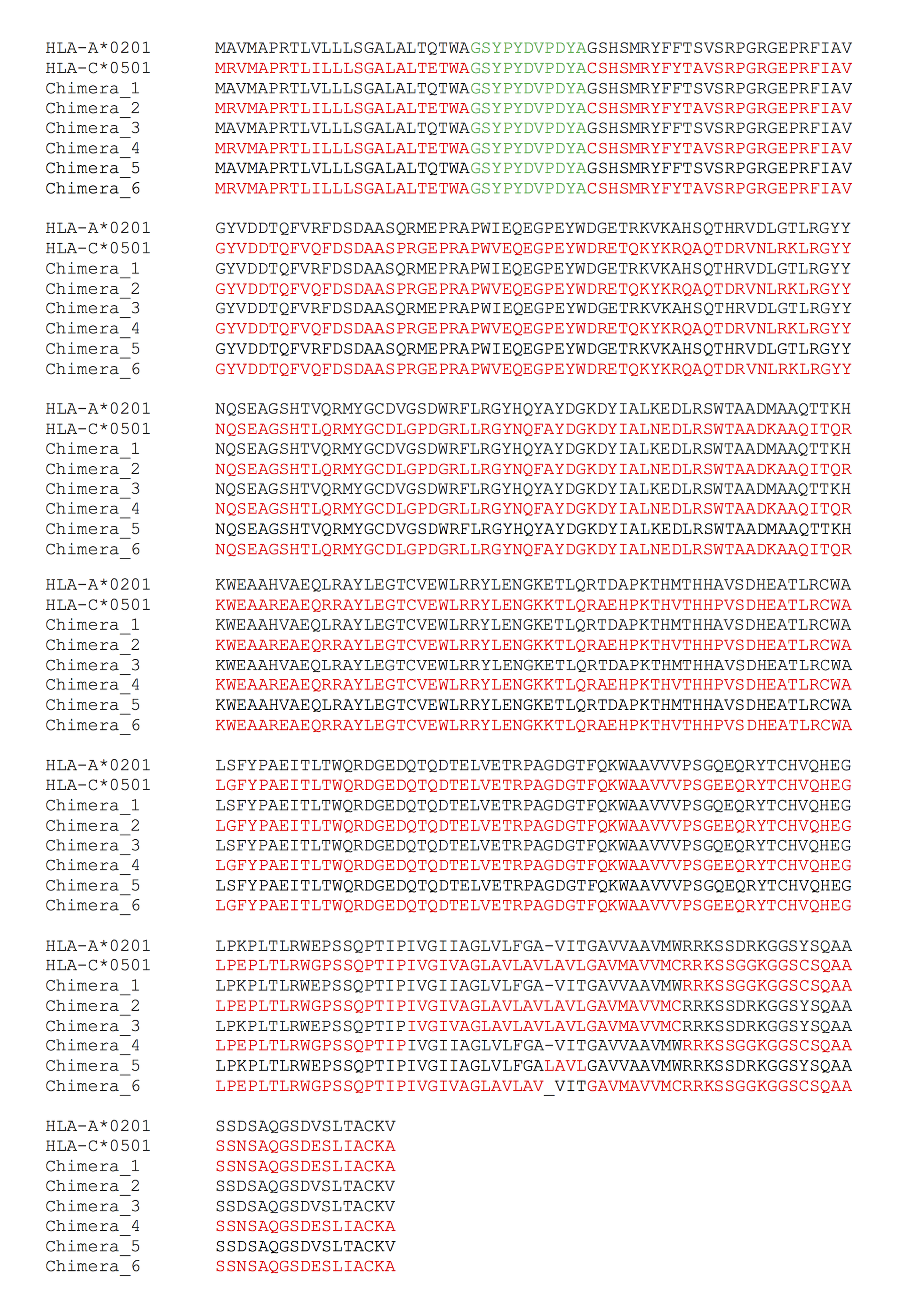

Supplement: S8 Fig — Black text indicates sequence from HLA-A, red indicates sequence from HLA-C, with green identifying the HA tag added at the N-terminus after the leader peptide. (TIF) [file ppat.1007257.s008.tif]

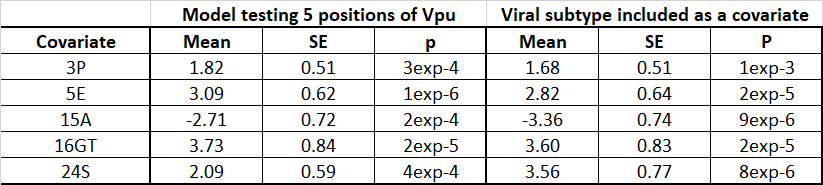

Supplement: S1 Table — Using 191 primary Vpu clones from chronic infection a multiple linear regression model identified residues at 5 positions of Vpu affecting HLA-C downregulation (Fig 4B, shown on the left). This analysis was repeated including viral subtype as a covariate (subtype A, B, C, or D) and all positions remain independently significant, shown on the right. (DOCX) [file ppat.1007257.s009.docx]

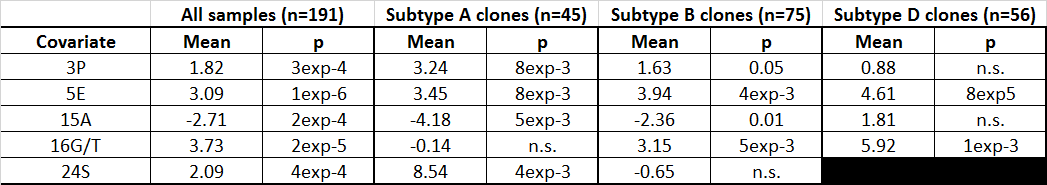

Supplement: S2 Table — Using 191 primary Vpu clones from chronic infection a multiple linear regression model identified residues at 5 positions of Vpu affecting HLA-C downregulation (Fig 4B, shown on the left). This analysis was repeated separately for infections with viruses of different subtype (shown right). Blacked box indicates n = 0. (DOCX) [file ppat.1007257.s010.docx]

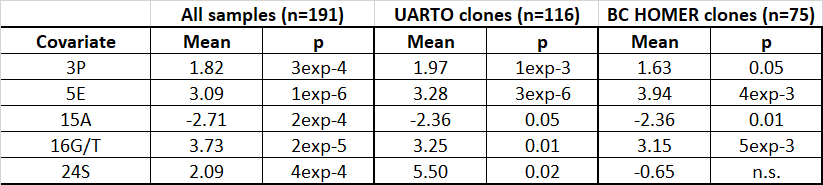

Supplement: S3 Table — Using 191 primary Vpu clones from chronic infection a multiple linear regression model identified residues at 5 positions of Vpu affecting HLA-C downregulation (Fig 4B, shown on the left). This analysis was repeated separately for individuals of each of the two cohorts used (shown right). (DOCX) [file ppat.1007257.s011.docx]

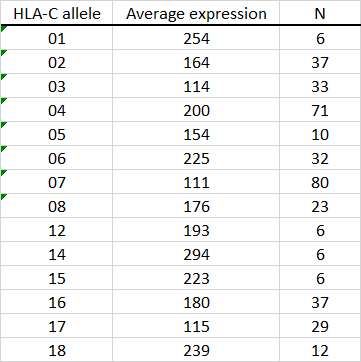

Supplement: S4 Table — The average expression level for each 2-digit HLA-C allele is reported here, from previously described staining of peripheral blood CD3+ cells from healthy donors analyzed by flow cytometry using the monoclonal antibody DT9 [31]. MFI of DT9 staining was plotted twice for each donor, once for each 2-digit HLA-C allele present, and the average MFI for each HLA-C allele is shown with N indicating the number of observations from which each average was determined. (DOCX) [file ppat.1007257.s012.docx]

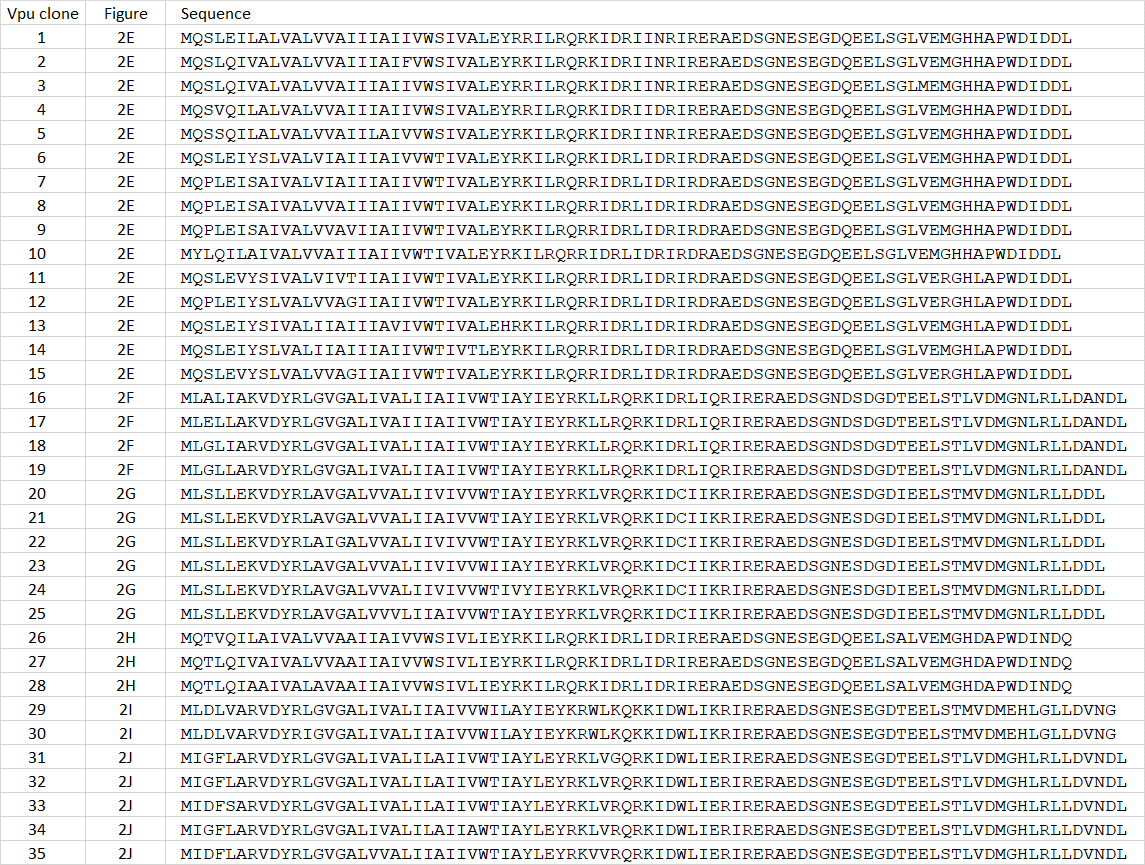

Supplement: S5 Table — (DOCX) [file ppat.1007257.s013.docx]

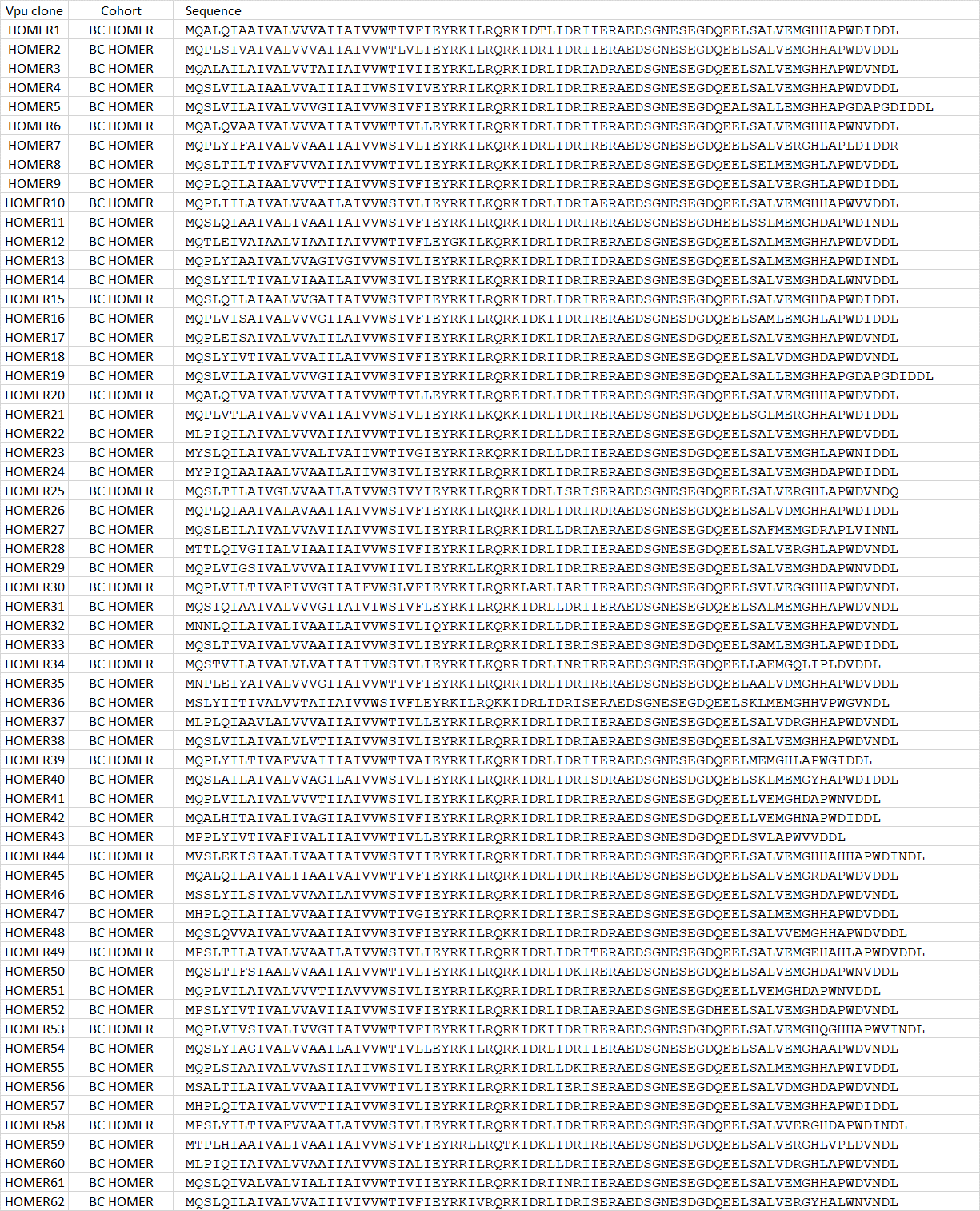


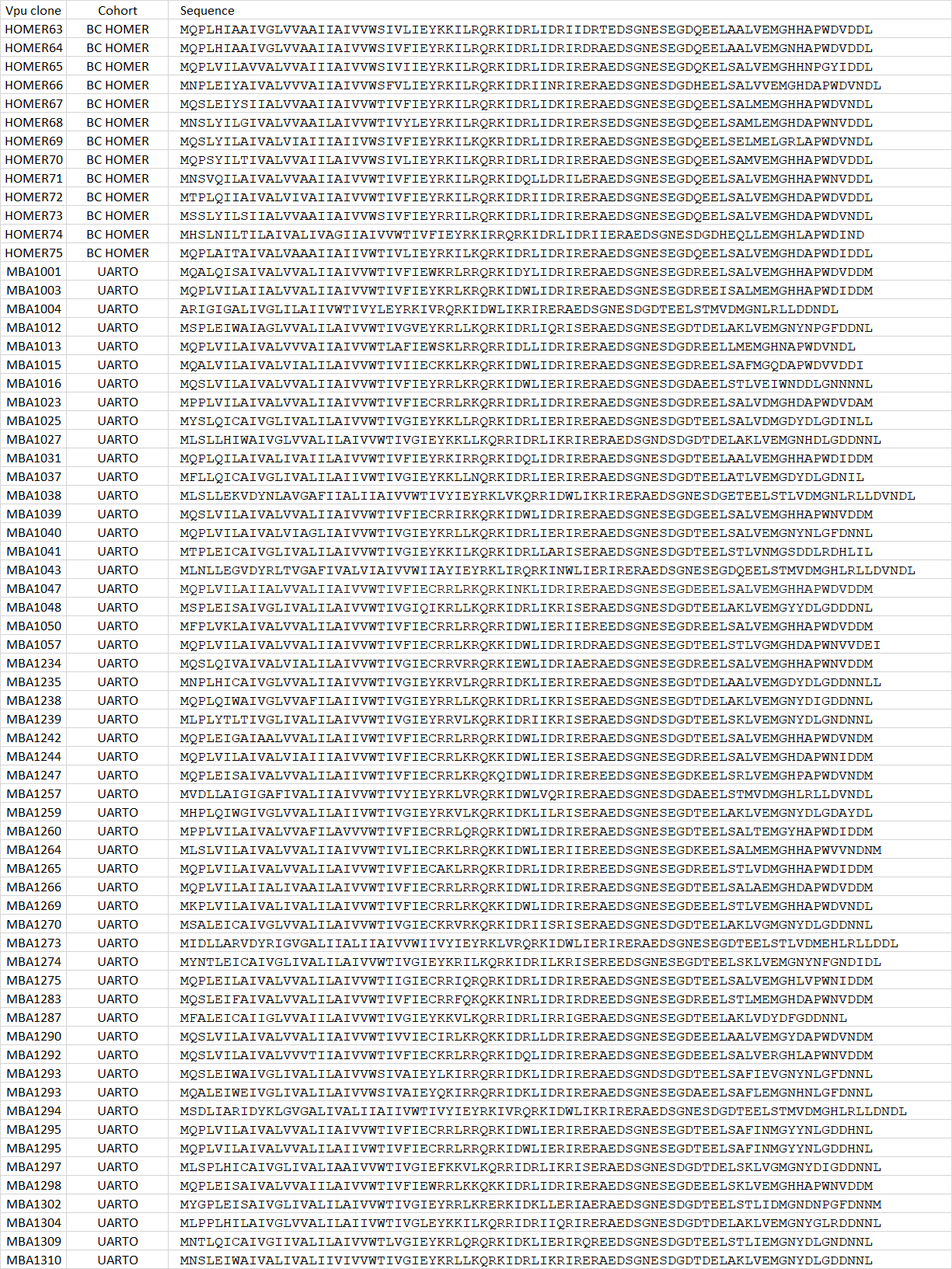


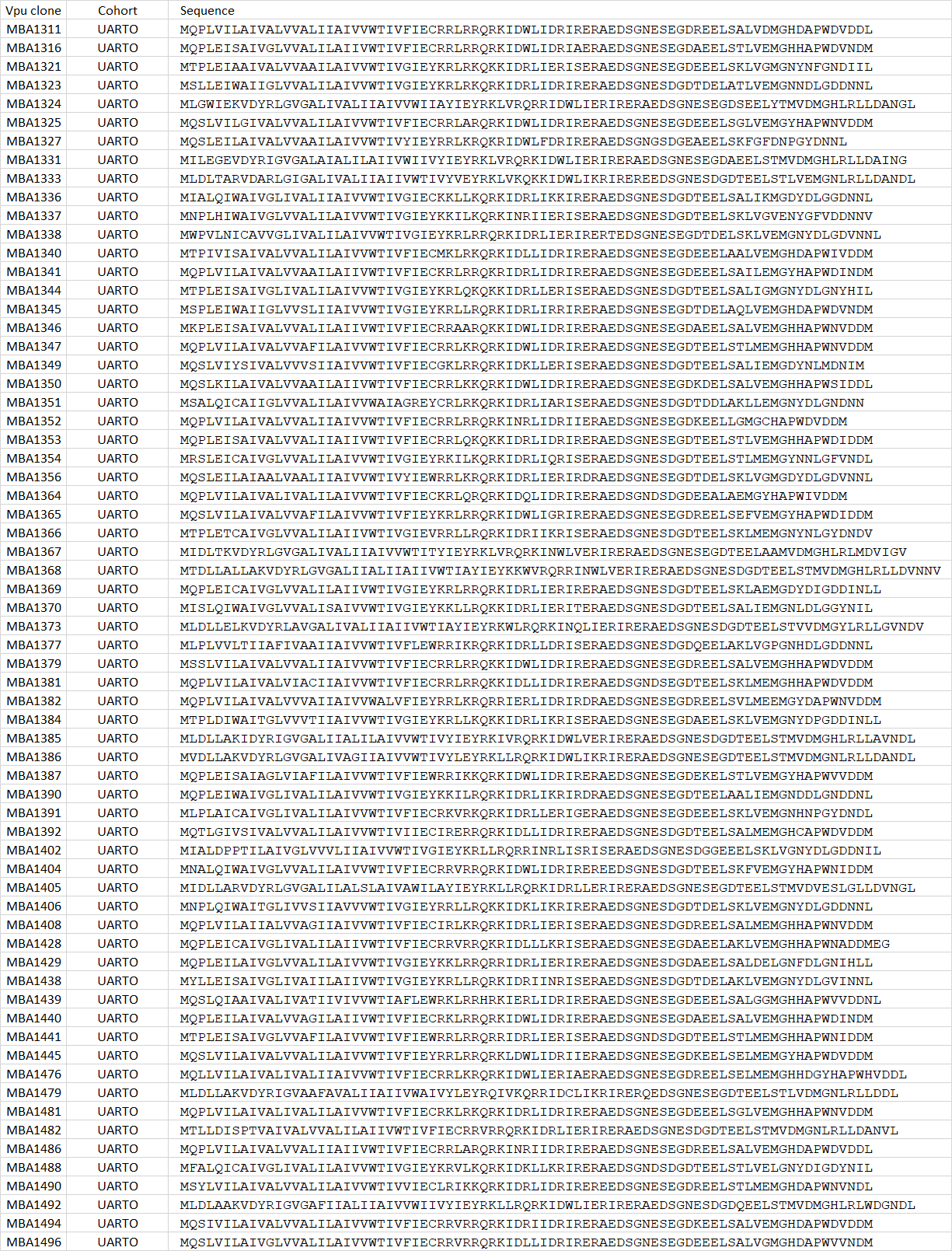

Supplement: S6 Table — (DOCX) [file ppat.1007257.s014.docx]
